# CHEK2 knockout is a therapeutic target for TP53-mutated hepatocellular carcinoma

**DOI:** 10.1038/s41420-023-01777-4

**Published:** 2024-01-19

**Authors:** Yuyan Chen, Zhengyi Zhu, Xingyu Wu, Hui Li, Wenxian Guan, Haozhen Ren

**Affiliations:** 1 Division of Hepatobiliary and Transplantation Surgery, Department of General Surgery, Nanjing Drum Tower Hospital, Affiliated Hospital of Medical School, Nanjing University, 210008 Nanjing, China; 2 Department of Radiology, Nanjing Drum Tower Hospital, Affiliated Hospital of Medical School, Nanjing University, 210008 Nanjing, China; 3 Department of General Surgery, Nanjing Drum Tower Hospital, Affiliated Hospital of Medical School, Nanjing University, 210008 Nanjing, China

**Keywords:** Target validation, Hepatocellular carcinoma

## Abstract

Currently, there is still a lack of novel and effective drug targets to improve the prognosis of hepatocellular carcinoma (HCC). Additionally, the role of CHEK2 in HCC has not been reported yet. The eQTLgen database and two HCC Genome-Wide Association Study (GWAS) datasets (ieu-b-4953, ICD10 C22.0) were used to find the drug target: CHEK2. Next, Colony, Edu, β-gal, and cell cycle analysis were facilitated to evaluate the role of CHEK2 knockout in HCC. In addition, Nultin-3 was added to evaluate the apoptosis of TP53-mutated HCC cells with CHEK2 knockout. Furthermore, MitoSox, electron microscopy, mitochondrial ATP, and NADH+/NADH levels were assessed in the CHEK2 knockout HCC cells with or without Metformin. Finally, cell-derived tumor xenograft was used to evaluate the role of CHEK2 knockout in vivo. We initially identified a potential drug target, CHEK2, through GWAS data analysis. Furthermore, we observed a significant upregulation of CHEK2 expression in HCC, which was found to be correlated with a poor prognosis. Subsequently, the results indicated that knocking out CHEK2 selectively affects the proliferation, cell cycle, senescence, and apoptosis of TP53-mutant HCC cells. Additionally, the introduction of Nultin-3 further intensified the functional impact on TP53-mutant cells. Then ClusterProfiler results showed high CHEK2 and TP53 mutation group was positively enriched in the mitochondrial ATP pathway. Then we used MitoSox, electron microscopy, mitochondrial ATP, and NADH + /NADH assay and found knockout of CHECK could induce the ATP pathway to inhibit the growth of HCC. Our research introduces a novel drug target for TP53-mutant HCC cells via mitochondrial ATP, addressing the limitation of Nultin-3 as a standalone treatment that does not induce tumor cell death.

## Introduction

Hepatocellular carcinoma (HCC) is the most common type of liver cancer and ranks as the fifth leading cause of cancer-related deaths worldwide [1]. The rising incidence of HCC highlights the pressing need for innovative therapeutic strategies, as the current drug treatments have limited efficacy. While drugs like sorafenib and lenvatinib have shown some success in improving patient prognosis, multicenter studies have revealed their limited impact on long-term survival due to treatment resistance and side effects [2]. Therefore, actively searching for novel and effective drug targets to enhance the prognosis of HCC patients is of crucial clinical importance.

The eQTLgen database serves as a large-scale research resource for studying the association between genetic regulatory loci known as expression quantitative trait loci (eQTL) and transcriptome expression in the human genome [3]. It aims to decipher the relationship between these eQTLs and RNA expression levels by integrating single-nucleotide polymorphism (SNP) information covering millions of loci across the human genome with RNA sequencing data from diverse tissues and cell types. Similarly, Mendelian randomization (MR) studies also utilize genetic variation in the form of SNPs as instruments for causal inference between exposures and outcomes, providing insights into whether observed associations are consistent with causal effects [4]. In MR studies, confounding bias can be minimized since genetic variations are inherently randomized at birth, ensuring that they are not influenced by external factors and reducing the possibility of reverse causation as genetic variants are determined before disease development [5]. By combining the approaches of eQTLgen and MR, we could potentially identify target genes for HCC treatment, as this joint analysis helps us explore potential causal relationships in a more robust manner. In recent years, significant advancements have been made in identifying drug targets for various diseases using the mentioned approach above. For instance, several studies have discovered that anti-lipid drugs like HMGCR and PCSK9 inhibitors can effectively reduce COVID-19-related outcomes [6]. Additionally, TYK2 has emerged as a potential drug target for multiple autoimmune disorders [7]. In this study, we selected two HCC Genome-Wide Association Study (GWAS) datasets (ieu-b-4953, ICD10 C22.0) and found that CHEK2 could be a potential drug target gene.

CHEK2, also known as Checkpoint kinase 2, is a critical protein kinase involved in the regulation of cell cycle progression [8]. It is involved in processes such as DNA double-strand breaks, DNA damage, and abnormal cell proliferation. When cells experience DNA damage, CHEK2 is activated and transmits signals by phosphorylating a series of substrates [9, 10]. This activation initiates the cell cycle checkpoint, halts cell cycle progression, and allows for DNA repair. Mutations in the CHEK2 gene have been found to be correlate with various cancers, particularly hereditary cancers like breast and colorectal cancer [11, 12]. Specifically, mutations in the CHEK2 gene can result in the loss or impairment of protein function, affecting DNA repair and cell cycle regulation mechanisms, thereby increasing the individual’s risk of developing cancer [13]. For instance, one of the common CHEK2 mutations associated with breast cancer is c.1100delC [14]. This mutation leads to a protein truncation and is significantly associated with genetic susceptibility to breast cancer. Research on CHEK2 in HCC is limited, with only a study indicating high expression of CHEK2 in HCC tissues [15]. However, the mechanisms by which CHEK2 affects the prognosis of HCC patients remain unclear.

## Results

### CHEK2 could be a potential drug target for HCC

**Fig. 1:**
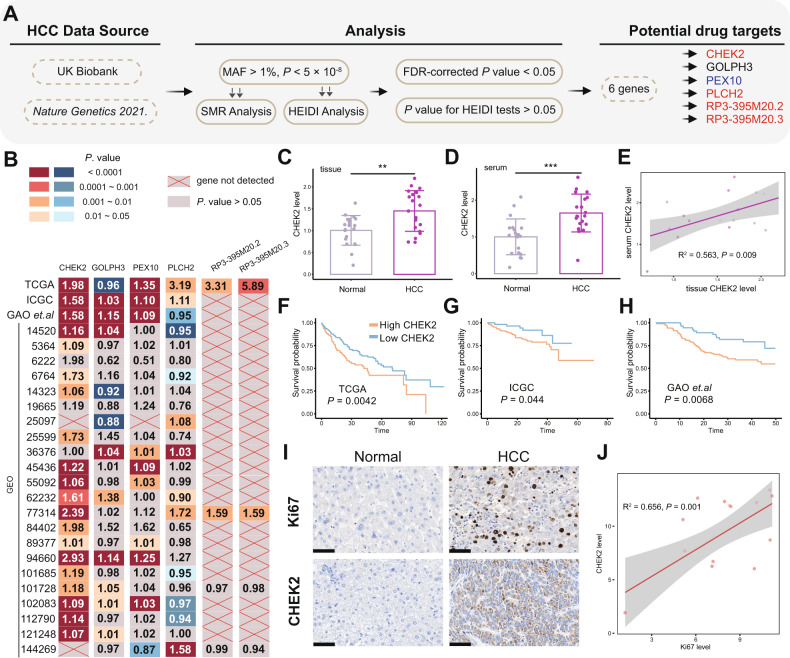
CHEK2 could be a potential drug target for HCC. **A** The workflow of identifying drug target genes. **B** Expression of several drug target genes in several HCC datasets. **C** The level of CHEK2 in the normal and HCC tissues. **D** The serum level of CHEK2 in the healthy donors and HCC samples. **E** The correlations between serum CHEK2 level and HCC CHEK2 level in 20 HCC samples. **F**–**H** KM analysis of low and high CHEK2 groups in the TCGA, ICGC, and GAO et al. databases. **I** The CHEK2 and Ki67 levels of IHC in the normal (*n* = 20) and HCC groups (*n* = 20). **J** The correlations between CHEK2 level and Ki67 level in 20 HCC samples.

To identify the potential HCC drug targets, we initially searched for two HCC GWAS datasets (ICD10 C22.0, ieu-b-4953) as outcome data and downloaded data from the eQTLgen database as reference data. Through SMR analysis, we identified 726 and 314 potential drug target genes based on the top SNPs in each dataset, respectively. Taking the intersection of these gene sets, we obtained six common genes (CHEK2, GOLPH3, PEX10, PLCH2, RP3-395M20.2, RP3-395M20.3). The workflow is illustrated in Fig. 1A and the SMR and MR analysis of these six genes are listed in Table 1. The results indicated CHEK2, PLCH2, RP3-395M20.2, and RP3-395M20.3 could be risk factors for HCC, while PEX10 could be a protective factor for HCC. Next, we investigated the expression levels of these potential target genes in both HCC and normal groups, we selected a total of 25 HCC expression datasets and found that CHEK2 exhibited significant upregulation in a majority of the HCC datasets (20/23), except for two datasets where it was not detected. However, the expression of other drug target genes in various HCC datasets was found to be inconsistent (Fig. 1B). Therefore, based on our observations, we hypothesize that CHEK2 may play a more significant role in HCC. Consequently, we have chosen to further investigate CHEK2 in our subsequent studies. The eQTLgen database we used was based on the SNP analysis of blood samples. Hence, we extracted both tissue and serum samples from 20 HCC patients and collected serum samples from 20 healthy individuals. We observed that the RNA levels of CHEK2 were significantly higher in both the serum and tissue samples of HCC patients compared to normal samples (Fig. 1C, D). Furthermore, between serum and tissue samples from the 20 pairs of HCC patients, we discovered a positive association in the expression of CHEK2. (Fig. 1E). Moreover, for investigating the clinical indices of CHEK2 in HCC, we selected TCGA, ICGC, and GAO et al. cohorts and we found higher CHEK2 group predicted poor overall survival rates than the lower CHEK2 group (Fig. 1F–H). In addition, in comparison to the low CHEK2 group, we found that the high CHEK2 group had worse disease-free interval (DFI), disease-specific survival (DSS), and progression-free interval (PFI) (sup Fig. 1A–C). Then, using IHC analysis, we discovered that the CHEK2 protein levels in the HCC group were significantly higher than those in the Normal group (Fig. 1I). Additionally, in HCC samples, there was a strong association between the IHC levels of CHEK2 and Ki67 (Fig. 1J).

**Table 1:** SMR association between expression of genes and HCC outcomes.

GWAS					eQTL			SMR			HEIDI	
Dataset	Genes	Beta	Se	*P*-value	Beta	Se	*P*-value	Beta	Se	*P*-value	*P*-value	nsnp
ieu−b−4953	CHEK2	0.26524	0.112938	0.0188469	0.0597826	0.00810063	<0.0001	4.43674	1.9825	0.02522383	0.2650548	20
	GOLPH3	1.44293	0.470894	0.00218231	−0.760142	0.0256463	<0.0001	−1.8982	0.62278	0.002303777	0.07860278	20
	PEX10	−0.2620	0.119524	0.0283323	0.132862	0.00979605	<0.0001	−1.9725	0.911291	0.03042141	0.07117842	20
	PLCH2	0.42379	0.142179	0.00287558	0.703135	0.0110791	<0.0001	0.60272	0.20243	0.002906472	0.1571539	20
	RP3-395M20.2	0.42379	0.142179	0.00287558	0.28422	0.0236051	<0.0001	1.4911	0.515344	0.003810954	0.4889532	17
	RP3-395M20.3	0.42379	0.142179	0.00287558	0.180591	0.0187417	<0.0001	2.34674	0.824108	0.004404904	0.5993256	9
ICD10 C22.0	CHEK2	0.00005	0.00001	0.00633099	0.0597826	0.00810063	<0.0001	0.00086	0.000117	<0.0001	1	20
	GOLPH3	−0.0001	0.000062	<0.00001	−0.189354	0.0118197	<0.0001	0.00067	0.000331	0.04263156	0.138445	8
	PEX10	−0.0001	0.000053	<0.00001	0.132862	0.00979605	<0.0001	−0.0011	0.000409	0.00384419	0.907163	20
	PLCH2	0.00014	0.000063	<0.00001	0.703135	0.0110791	<0.0001	0.00020	0.000089	0.01967015	0.950303	20
	RP3-395M20.2	0.00014	0.000063	<0.00001	0.28422	0.0236051	<0.0001	0.00051	0.000225	0.02193467	0.2166663	12
	RP3-395M20.3	0.00014	0.000063	<0.00001	0.180591	0.0187417	<0.0001	0.00081	0.000359	0.0232958	0.2713227	8

### Knockout of CHEK2 selectively induces proliferation arrest, cell cycle blockade, and senescence in HCC cells with TP53 mutation

**Fig. 2:**
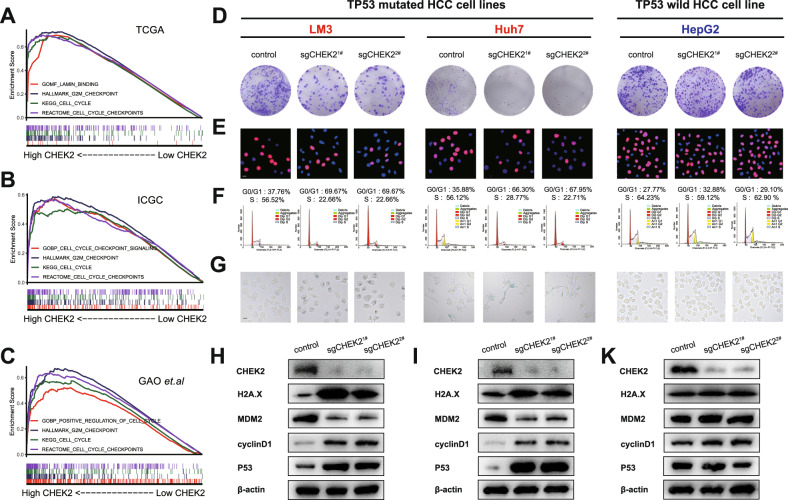
Knockout of CHEK2 selectively induces proliferation arrest, cell cycle blockade, and senescence in HCC cells with TP53 mutation. **A**–**C** Single-gene GSEA of low and high CHEK2 groups in the TCGA, ICGC, and GAO et al. databases. **D**–**G** Colony, Edu, cell cycle and β-gal staining of control, sgCHEK2^1#^ and sgCHEK2^2#^ groups in LM3, Huh7, and hepG2 cell lines (*n* = 3). **H**–**K** Western blot results of control, sgCHEK2^1#^ and sgCHEK2^2#^ groups in LM3, Huh7, and hepG2 cell lines.

To elucidate the role of CHEK2 in HCC, we performed single-gene GSEA for CHEK2 using three databases: TCGA, ICGC, and GAO et al. We observed the enrichment of CHEK2 in multiple cell cycle functions (Fig. 2A–C). Interestingly, CHEK2 also showed enrichment in Lamin binding, which is a marker of senescence. Therefore, we validated the expression of CHEK2 in five HCC cell lines (97H, LM3, BEL-7404, Huh7 and hepG2) and observed relatively higher expression of CHEK2 in LM3, Huh7, and HepG2 cell lines (sup Fig. 2A). We initiated plate cloning and Edu experiments as our initial investigations and we found inhibition of CHEK2 could inhibit the cell proliferation of LM3 and Huh7 cells, while the proliferation capacity of HepG2 cells was found to be unaffected by CHEK2 expression (Fig. 2D, E; sup Fig. 2B–D). Then, through the cell cycle and senescence experiments, we discovered that inhibiting CHEK2 significantly induced G0 phase arrest and promoted senescence in LM3 and Huh7 cells. However, HepG2 cell cycle or senescence functionality was unaffected by CHEK2 suppression (Fig. 2F, G; sup Fig. 2E–G). Further, western blot analysis confirmed the previous findings as well (Fig. 2H–K). Based on previous studies, it was indicated that LM3 and Huh7 are HCC cell lines with TP53 mutation, while HepG2 is a TP53 wild-type HCC cell line. Therefore, we hypothesize that the knockout of CHEK2 selectively induces proliferation arrest, cell cycle blockade, and senescence in HCC cells with TP53 mutation.

### Combining Nultin-3 further induces cell cycle arrest and inhibits growth in CHEK2-inhibited HCC cells with TP53 mutation

**Fig. 3:**
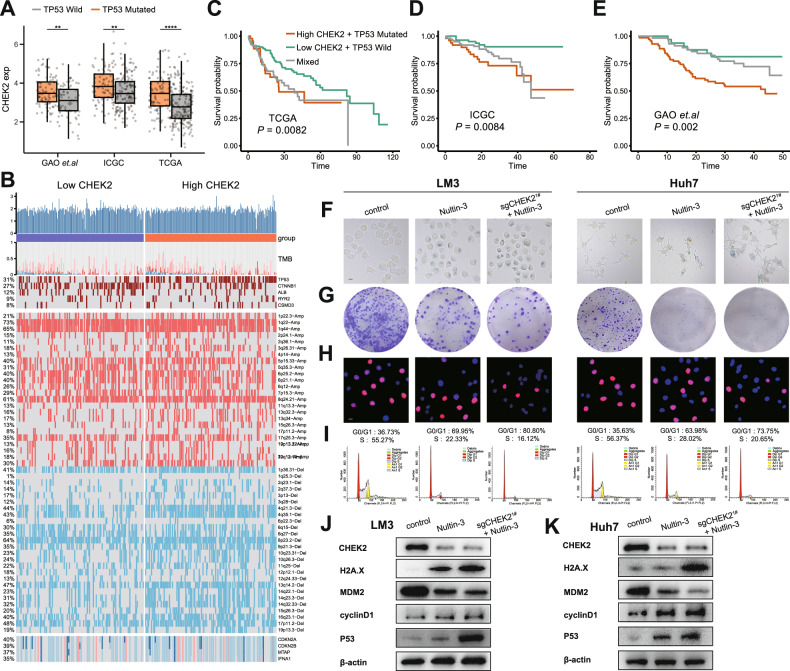
Combining Nultin-3 further induces cell cycle arrest and inhibits growth in CHEK2-inhibited HCC cells with TP53 mutation. **A** Expression of CHEK2 between the TP53 wild and mutation groups in the TCGA, ICGC, and GAO et al. databases. **B** Landscape of genomic mutations and CNV data between the low CHEK2 and high CHEK2 groups in HCC. **C**–**E** KM analysis of high CHEK2/TP53 mutation, low CHEK2/TP53 wild, and mixed groups in the TCGA, ICGC, and GAO et al. databases. **F**–**I** Colony, Edu, cell cycle and β-gal staining of control, Nultin-3, and sgCHEK2^1#^ + Nultin-3 groups in LM3, Huh7 cell lines (*n* = 3). **J**, **K** Western blot results of control, sgCHEK2^1#^ and sgCHEK2^1#^ + Nultin-3 groups in LM3, Huh7 cell lines.

To further investigate the relationship between CHEK2 and TP53-mutated HCC cells, we analyzed data from three cohorts (TCGA, ICGC, and GAO et al.) and found a significant upregulation of CHEK2 expression in tissues with TP53 mutations (Fig. 3A). Additionally, we compared the genomes of the groups with low and high CHEK2 levels. Our analysis revealed a higher frequency of TP53 mutations in the high CHEK2 group. Furthermore, we observed increased frequencies of copy number variants (CNV) in the high CHEK2 group compared to the low CHEK2 group across various chromosomal arms (Fig. 3B). To assess the correlation between TP53 mutation, CHEK2 expression, and patient survival, we generated a Kaplan-Meier survival curve. Interestingly, patients with high CHEK2 expression and TP53 mutation had a worse prognosis compared to other subgroups (Fig. 3C–E). Hence, we hypothesize that simultaneous inhibition of TP53 mutation and knockout of CHEK2 may further suppress cell cycle progression, proliferation et al. in HCC with TP53 mutation. We then selected Nultin-3, which has been investigated as a potential therapeutic agent specifically targeting TP53-mutated cancer cells [16]. Furthermore, we further supplemented with 10 μM Nultin-3 in the previous knockout of CHEK2 HCC cell lines (LM3, Huh7). Subsequently, we performed cloning formation, Edu assay, β-galactosidase staining, and cell cycle experiments. We observed that the addition of Nultin-3 could induce a more pronounced arrest of HCC cells in the G0/G1 phase, inhibiting proliferation, and further promoting senescence (Fig. 3F–I; sup Fig. 3A–D). Furthermore, western blot analysis also confirmed the previous findings (Fig. 3J, K).

### Knockout of CHEK2 triggers apoptosis in Nultin-3-treated HCC cells

**Fig. 4:**
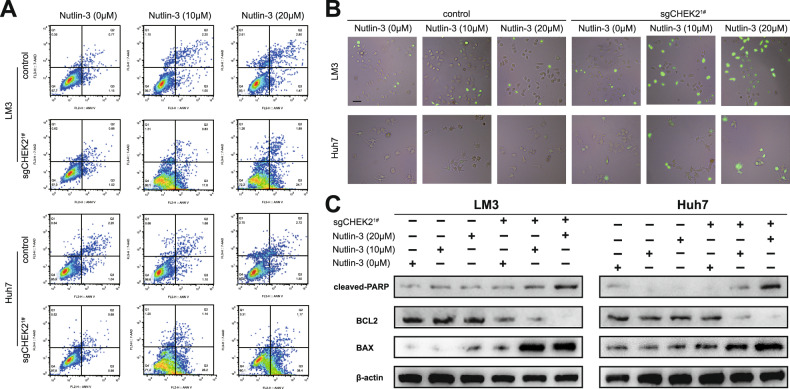
Knockout of CHEK2 triggers apoptosis in Nultin-3-treated HCC cells. **A** Apoptosis analysis of control and sgCHEK2^1#^ groups with 0, 10, 20 μM Nutlin-3 in LM3, Huh7 cell lines (*n* = 3). **B** Caspase-3 staining of control and sgCHEK2^1#^ groups with 0, 10, 20 μM Nutlin-3 in LM3, Huh7 cell lines (*n* = 3). **C** Western blot results of control and sgCHEK2^1#^ groups with 0, 10, 20 μM Nutlin-3 in LM3, Huh7 cell lines.

Research suggests that Nutlin-3-mediated p53 activation can induce reversible tumor cell cycle arrest, while without causing cell apoptosis [17]. We increased the concentration gradient from the standard 10 μM and observed that a single treatment with Nutlin-3 did not show significant changes in TP53-mutated HCC cell lines, as assessed by Annexin-V and 7-AAD double staining. Interestingly, we found that knocking out CHEK2 in HCC cell lines resulted in increased apoptosis, particularly in the early stages of apoptosis (Fig. 4A; sup Fig. 4A, B). Furthermore, we validated the above results by assessing the caspase-3 green apoptosis-assay reagent. We found increased apoptosis in HCC cells with CHEK2 knockout (Fig. 4B). Additionally, western blotting results confirmed these findings (Fig. 4C). Hence, knockout of CHEK2 could trigger apoptosis in Nultin-3 treated HCC cells.

### Combining Nultin-3 and knockout of CHEK2 exacerbates the loss of mitochondrial ATP in HCC

**Fig. 5:**
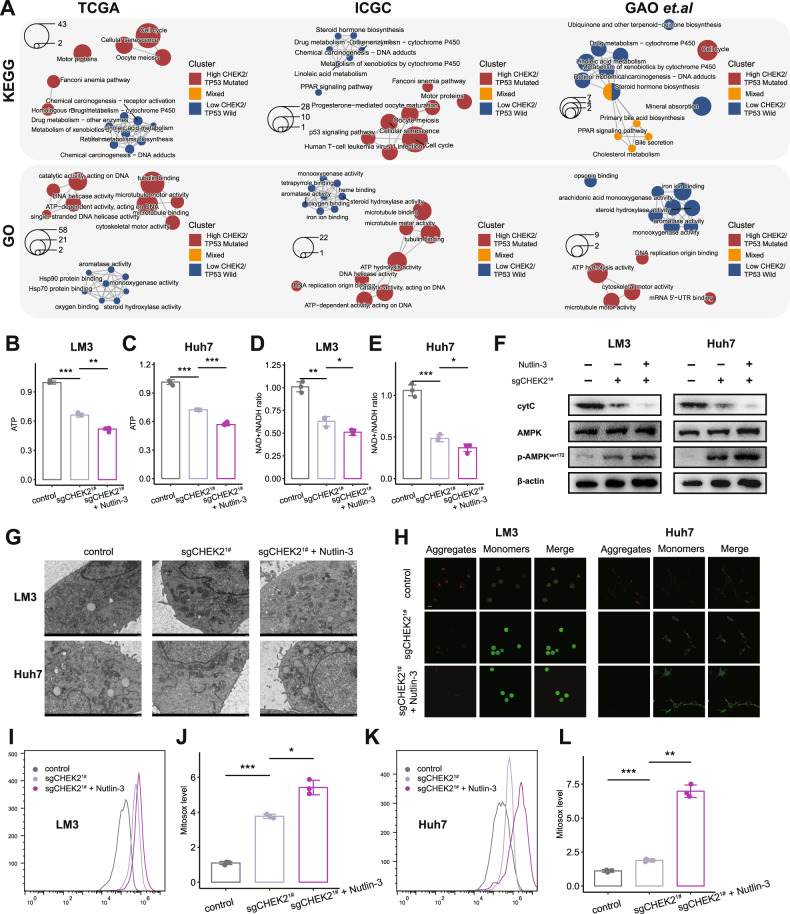
Combining Nultin-3 and knockout of CHEK2 exacerbates the loss of mitochondrial ATP in HCC. **A** ClusterProfiler results of high CHEK2/TP53 mutation, low CHEK2/TP53 wild, and mixed groups in the TCGA, ICGC, and GAO et al. databases. **B**–**E** ATP level and NADH+/NADH ratio of control, sgCHEK2^1#^ and sgCHEK2^1#^ + Nultin-3 groups in LM3, Huh7 cell lines. **F** Western blot results of control, sgCHEK2^1#^ and sgCHEK2^1#^ + Nultin-3 groups in LM3, Huh7 cell lines. **G**–**L** transmission electron microscopy, JC1 and MitoSox level of control, sgCHEK2^1#^ and sgCHEK2^1#^ + Nultin-3 groups in LM3, Huh7 cell lines (*n* = 3).

To further investigate the potential pathways linking CHEK2 and TP53-mutated HCC cells, we initially divided samples into three groups in TCGA, ICGC, and GAO et al. databases: high CHEK2/TP53 mutation, low CHEK2/TP53 wild, and mixed. Subsequently, the Kyoto Encyclopedia of Genes and Genomes (KEGG) and Gene Ontology (GO) enrichment analyses were performed. In the results, we consistently found that the high CHEK2/TP53 mutation group was associated with cell cycle across all three databases, which aligns with our previous findings. Interestingly, we also discovered that the high CHEK2/TP53 mutation group exhibited enrichment in ATP-related pathways (Fig. 5A). The synthesis of ATP primarily occurs through the chemical reactions of oxidative phosphorylation within the mitochondria [18]. High-energy electrons must be transferred from carriers like NADH to respiratory chain complexes found in the inner mitochondrial membrane in order for this process to proceed. Further, we observed a significant decrease in ATP levels and NADH+/NADH ratio upon knocking out CHEK2. Furthermore, the addition of Nutlin-3 further intensified this process (Fig. 5B–E). Adenosine Monophosphate-Activated Protein Kinase (AMPK) acts as a critical cellular energy sensor and regulator. When cellular ATP levels decrease, AMPK is activated [19]. Hence, we have also validated this point through western blotting (Fig. 5F). In addition, Consistent with previous findings, we further investigated the mitochondrial state using transmission electron microscopy. We observed that the combined knockout of CHEK2 and treatment with Nutlin-3 promoted mitochondrial swelling and loss of cristae, as detected by transmission electron microscopy (Fig. 5G). In addition, we used the JC1 method to assess mitochondrial membrane potential. Our results demonstrated that the combined knockout of CHEK2 and treatment with Nutlin-3 further induced the dissipation of mitochondrial membrane potential (Fig. 5H). Dysfunctional electron transport in mitochondria could also lead to an increase in mitochondrial membrane potential (Δψm), which, in turn, promotes the generation of ROS [20]. This relationship between mitochondrial membrane potential and ROS production has been established in previous studies [21]. Finally, combined knockout of CHEK2 and treatment with Nutlin-3 further induced the generation of MitoSox level (Fig. 5I–L).

### Combining Nultin-3 and knockout of CHEK2 could influence several biological processes of HCC via mitochondrial ATP

**Fig. 6:**
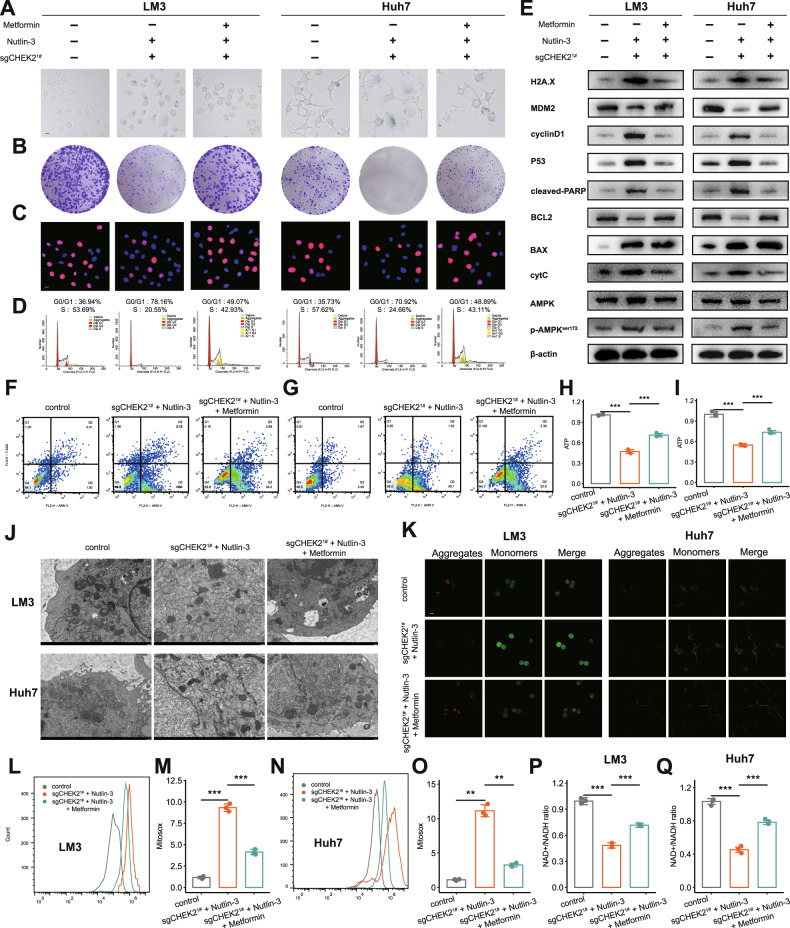
Combining Nultin-3 and knockout of CHEK2 could influence several biological processes of HCC via mitochondrial ATP. **A**–**D** β-gal staining, colony, Edu and cell cycle of control, sgCHEK2^1#^ + Nultin-3 and sgCHEK2^1#^ + Nultin-3 + Metformin groups in LM3, Huh7 cell lines (*n* = 3). **E**–**G** Western blot results and apoptosis assays of control, sgCHEK2^1#^ + Nultin-3 and sgCHEK2^1#^ + Nultin-3 + Metformin groups in LM3, Huh7 cell lines. **H**–**Q** ATP level, NADH+/NADH ratio, transmission electron microscopy, JC1 and MitoSox level of control, sgCHEK2^1#^ + Nultin-3 and sgCHEK2^1#^ + Nultin-3 + Metformin groups in LM3, Huh7 cell lines (*n* = 3).

To further investigate whether HCC cells treated with knockout of CHEK2 and Nutlin-3 could affect various biological processes in HCC through the mitochondrial ATP pathway, we selected Metformin, which acts on several cellular pathways to enhance ATP production in tumor cells via inhibiting complex I of the mitochondrial electron transport chain [22]. From functional assays, we observed treatment of Metformin could reverse the cellular senescence, cell cycle arrest, reduced cell proliferation, and increased apoptosis of HCC cells combined with CHEK2 knockout and Nutlin-3 treatment (Fig. 6A–D; sup Fig. 5A–D). Next, we facilitated western blot analysis to prove these results (Fig. 6E). In addition, treatment of Metformin could also reverse the apoptotic HCC cells (Fig. 6F, G; sup Fig. 5E, F). Finally, we further confirmed that Metformin can effectively restore mitochondrial dysfunction and decrease in mitochondrial ATP levels caused by combined CHEK2 knockout and Nutlin-3 treatment (Fig. 6H–Q). The results were consistent with our expectations. Hence, combining Nultin-3 and knockout of CHEK2 could influence several biological processes of HCC via the mitochondrial ATP pathway.

### Inhibition of CHEK2 suppresses HCC proliferation in vivo

**Fig. 7:**
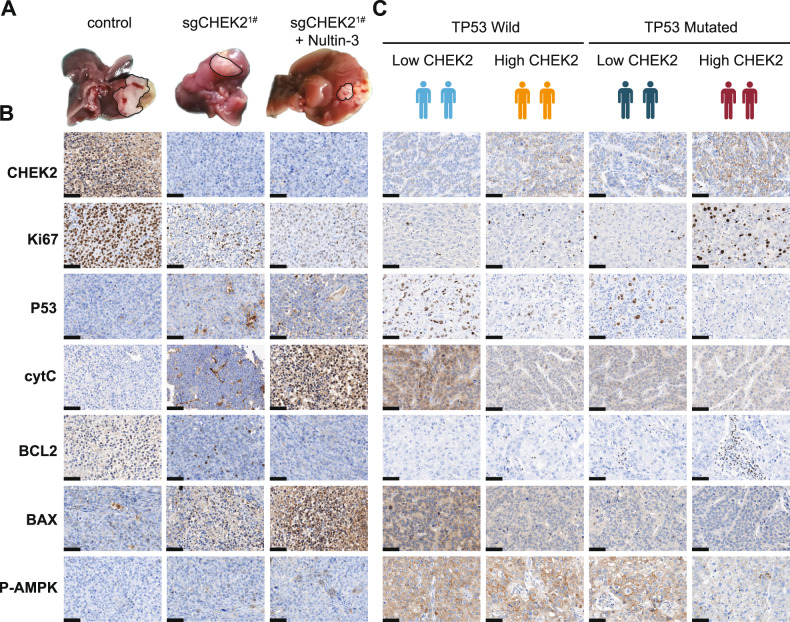
Inhibition of CHEK2 suppresses HCC proliferation in vivo. **A** Construction of CDTX model for HCC between the control, sgCHEK2^1#^ and sgCHEK2^1#^ + Nultin-3 groups (*n* = 5). **B** IHC results of CDTX model for HCC between the control, sgCHEK2^1#^ and sgCHEK2^1#^ + Nultin-3 groups (*n* = 5). **C** IHC results of TP53 mutation and TP53 wild HCC slices.

To learn more about the function of mice with CHEK2 deletion, we established a CDTX model of HCC. Our findings revealed that knocking out CHEK2 can suppress the development of HCC, and this effect was intensified when Nultin-3 was introduced (Fig. 7A). Subsequently, we conducted IHC analysis and observed that P53, cytC, BAX, and phosphorylated AMPK levels increased in the presence of Nultin-3 and CHEK2 deletion, but Ki67 and BCL2 expression was decreased (Fig. 7B). Finally, we selected human tissue sections from both TP53-mutant and TP53 wild-type HCC samples and observed consistent results with our previous findings (Fig. 7C). Overall, our results showed combination of Nultin-3 and CHEK2 knockout appears to have a greater potential for inhibiting HCC through the mitochondrial ATP pathway.

## Discussion

Currently, the clinical management of HCC primarily relies on surgical resection and chemotherapy, but these approaches have limitations and often yield unsatisfactory outcomes [23]. For the treatment of advanced HCC, only the multi-kinase inhibitors sorafenib and lenvatinib have received approval [24]. Regorafenib, a different multi-kinase inhibitor, and nivolumab, an anti-PD-1 antibody, have just come to light as new-generation medications for the treatment of advanced HCC [25]. The prognosis for patients is still poor in spite of these developments. Additionally, there are currently few alternatives for druggable targets in preclinical research for HCC therapy. Finding more possible targets that can be successfully addressed for treatment is therefore urgently needed. The current gold standard for determining disease inference causal links is randomized controlled trials (RCTs). However, implementing RCTs can be challenging due to ethical restrictions [26]. MR is an alternative approach that can serve as a substitute for RCT studies, and its evidence level is only slightly lower than that of RCTs. Therefore, using the eQTLgen database and MR methods, we have identified six potential drug targets (CHEK2, GOLPH3, PEX10, PLCH2, RP3-395M20.2, RP3-395M20.3) for HCC. Finally, based on the significant upregulation of CHEK2 gene expression in 25 HCC databases, we have selected CHEK2 as the focus of our subsequent research. Despite being considered a tumor suppressor gene in colorectal and breast cancer, CHEK2 can paradoxically function as a potential oncogene in HCC, as our research findings suggest. In line with our study, Wu et al. have also reported similar conclusions [15]. This discrepancy may be attributed to the distinct tumor microenvironment in HCC compared to the other two types of cancer, which could contribute to the significantly lower postoperative survival rate observed in HCC. Therefore, understanding the functional role of the potential drug target CHEK2 is crucial for improving postoperative prognosis in HCC patients.

To mimic the CRISPR technology of drug targets, we constructed two sgCHEK2 sequences. Interestingly, we observed that the knockout of CHEK2 in LM3 and Huh7 cell lines resulted in decreased proliferation capacity and increased cellular senescence. Additionally, more HCC cells were arrested in the G0/G1 phase. However, the knockout of CHEK2 in the hepG2 cell line had no significant impact. Some studies revealed that LM3 and Huh7 cell lines harbor TP53 mutations, while hepG2 is a TP53 wild-type cell line. TP53 mutations are among the most common genetic abnormalities in HCC [27]. Studies have shown a high prevalence of TP53 mutations in HCC patients, especially in advanced stages of HCC [28]. TP53 mutations could also promote HCC cell proliferation, invasion, and metastasis while inhibiting apoptosis and DNA repair, thereby accelerating tumor progression and typically indicating a poor prognosis [29]. Previous studies by Wang et al. have shown that inducing senescence in TP53-mutant liver cancer cells, followed by targeted elimination of these senescent cells using specific drugs, can improve patient survival [30]. However, it’s crucial to understand that senescent and apoptotic cells are not the same thing. Senescent cells may release a variety of interleukins and inflammatory substances known as the SASP (senescence-associated secretory phenotype), which can further induce proliferation and metastasis of HCC cells [31]. Overall, HCC patients with TP53 mutations have a poorer prognosis, and currently, the Food and Drug Administration (FDA) has not approved any drugs specifically targeting TP53 mutations. Therefore, our research findings, indicating that knockout of CHEK2 selectively weakens the relevant biological functions of HCC cells with TP53 mutations, may provide a potential target for TP53-mutant HCC cells.

Research indicates that under normal conditions, MDM2 binds to p53 and promotes its degradation, thus inhibiting p53 activity [32]. However, Nutlin-3 is a compound that inhibits the interaction between p53 and MDM2, resulting in increased stability and activity of p53 [16]. When applied to tumor cells, Nutlin-3 can restore the tumor-suppressive function of p53 and induce cell cycle arrest. Although Jiang et al. found that Nutlin-3 can regulate p53-mediated ferroptosis in human cancer cells, it is generally believed that Nutlin-3 does not induce cell death, which may limit its effectiveness in cancer treatment [17]. Interestingly, our study discovered that knockout of CHEK2 not only further enhances cell cycle arrest but may also act as a switch to initiate apoptosis in response to Nutlin-3. This provides broader prospects for the use of Nutlin-3 in treating TP53-mutant HCC.

In this study, we identified significant enrichment of HCC cells with TP53 mutations and high expression of CHEK2 in the mitochondrial ATP pathway. Mitochondrial ATP plays a crucial role in tumors [33]. As the primary supplier of cellular energy, mitochondria generate the majority of ATP through oxidative phosphorylation. Tumor cells have high-energy demand due to their rapid proliferation, leading to an increase in mitochondrial numbers and enhanced respiratory chain activity [34]. By supplying sufficient ATP, mitochondria support tumor cell growth, division, and invasion. However, when mitochondrial function is impaired in tumor cells, ATP production decreases, resulting in metabolic disturbances and the occurrence of phenomena such as mitochondrial membrane depolarization, reduced activity of respiratory chain complexes, or increased lactate production (known as the Warburg effect). For example, Daglish et al. found that IMT1B directly inhibits mitochondrial DNA transcription and drastically lowers ATP synthesis in mitochondria by targeting human mitochondrial RNA polymerase (POLRMT), which is overexpressed in many cancer cells [35]. Furthermore, the downregulation of mitochondrial ATP is involved in the regulation of programmed cell death, known as apoptosis. Mitochondria release cytochrome c and other apoptotic proteins, activating apoptotic pathways, suppressing tumor cell survival, and exerting antitumor effects. For instance, Lv et al. discovered that Ainsliadimer A binds to cysteine 173 of PRDX1 and methionine 172 of PRDX2, thereby triggering the mitochondrial apoptotic pathway and ultimately inhibiting colon cancer development [36]. In addition, we found that in HCC cell lines, knocking out CHEK2 can change the potential and permeability of the mitochondrial membrane. Most solutes cannot pass freely through the mitochondrial inner membrane because it is a lipid bilayer structure with extremely selective permeability. The ATP concentration gradient between the cytoplasm and mitochondria is maintained by particular transport proteins on the mitochondrial inner membrane that control the exchange of ATP and ADP. One such protein is the ADP/ATP translocase [37]. Therefore, we speculate that knocking out CHEK2 in HCC cells may increase the permeability of the mitochondrial inner membrane, leading to the release of ATP from mitochondria into the cytoplasm. This process may play a role in activating apoptotic pathways, such as the important marker cytC [38].

In this study, we initially identified the drug target CHEK2 for HCC through MR screening and found that CHEK2 knockout selectively induces growth arrest in TP53-mutant cells. Additionally, we observed that the combination of Nultin-3 and CHEK2 knockout is more likely to inhibit HCC through the mitochondrial ATP pathway. However, there are limitations in this study. Firstly, we haven’t fully understood how CHEK2 knockout affects Nultin-3 and ultimately triggers the mitochondrial apoptotic pathway. To better understand their connection, further research is required. Secondly, mitochondrial function has a dual nature, and some studies suggest that impaired mitochondrial function can make tumor cells more adaptable to hypoxic environments and promote cell proliferation, metastasis, and drug resistance. Although our study mainly focuses on mitochondrial ATP and the apoptotic pathway to explain this phenomenon, the metabolic environment disruption associated with HCC needs further exploration. Overall, our research introduced a putative target for TP53-mutant HCC cells and overcomes the limitation of Nultin-3 alone, which fails to induce tumor cell death.

## Methods

### Design and ethics of MR study

Figure 1 A illustrates the design of the MR study. The study utilized publicly available data from a comprehensive genome-wide association study on blood expression, which can be found at the page (https://eqtlgen.org/cis-eqtls.html). Two datasets from HCC GWAS were incorporated into this analysis. The UK Biobank dataset (ID: ieu-b-4953), encompassing 372,184 European samples and 6,304,034 SNPs, was obtained from the website (https://gwas.mrcieu.ac.uk/datasets/ieu-b-4953/). Additionally, data from Jiang et al. (ID: ICD10 C22.0) was acquired from the website (https://www.ebi.ac.uk/gwas/efotraits/EFO_0000182), featuring 456,348 European samples [39]. All included studies have received approval from their respective ethical review committees.

### MR analysis and potential drug targets selecting

We conducted a robust two-sample MR analysis using eQTL index SNPs to examine the association between eQTL and HCC. To enhance the validity of our findings, we employed Summary-data-based Mendelian Randomization (SMR) analysis, which is based on summary data, to identify potential drug targets for HCC. The SMR test provided significance levels, allowing us to assess the strength of associations [40]. Furthermore, we utilized the Heterogeneity in Dependent Instrument (HEIDI) test, which offers improved accuracy compared to other methods involving GWAS and molecular eQTL data, to distinguish pleiotropic models from linkage models. Associations with a P-value less than 0.05 in the HEIDI test were considered likely to be influenced by pleiotropy and thus excluded from further analysis. We performed the SMR analysis using the SMR software tool (version 1.3.1). The procedure was according to Yang et al. reported (https://yanglab.westlake.edu.cn/software/smr/#SMR&HEIDIanalysis).

### Collection and processing of expression profiles and clinical data

To detect the gene expression levels of six specific genes (CHEK2, GOLPH3, PEX10, PLCH2, RP3-395M20.2, RP3-395M20.3) in both HCC and normal tissues, we utilized various public datasets. These included the Cancer Genome Atlas (TCGA), International Cancer Genome Consortium (ICGC), GAO et al cohorts [41, 42], as well as 22 Gene Expression Omnibus database (GEO) datasets (GSE5364, GSE6222, GSE6764, GSE14323, GSE14520, GSE19665, GSE25097, GSE25599, GSE36376, GSE45436, GSE55092, GSE62232, GSE77314, GSE84402, GSE89377, GSE64990, GSE101685, GSE101728, GSE102083, GSE112790, GSE121248, GSE144269). For clinical data, we relied on the TCGA, ICGC, and GAO et al. cohorts [43]. The data from the TCGA-LIHC database was downloaded from the website (https://portal.gdc.cancer.gov). The data from the ICGC-LIRI-JP database was obtained from the website (https://dcc.icgc.org/). Meanwhile, the data from several GEO databases were downloaded from the website (https://www.ncbi.nlm.nih.gov/geo/). Additionally, we extracted detailed clinical information about the Gao et al. cohort (OEP000321) directly from the supplementary files of the respective paper, while the expression profiles were accessed from the website (https://ngdc.cncb.ac.cn/).

### Patients and sample collection

This study obtained ethical approval from the Nanjing Drum Tower Hospital Ethics Committee. The patients were divided into two cohorts. Cohort 1 consisted of 20 HCC patients who underwent hepatectomy. For each HCC patient, an HCC specimen, a matched normal specimen, and a fresh plasma sample before surgery were collected and analyzed. Furthermore, serum samples were obtained from 20 healthy individuals. Further, these 20 samples were paraffin-embedded into slides.

### Cell culture

LM3 (human HCC cell line), Huh7 (human HCC cell line), and HepG2 (human HCC cell line) were purchased from Genechem (Shanghai, China), which were cultured in DMEM medium mixed with 10% FBS.

### RNA extraction and qRT-PCR analysis

Total RNA was extracted from tissue and serum samples of 20 patients using Trizol reagent (Ambion, Austin, TX, USA). Subsequently, cDNA synthesis was performed using a first-strand cDNA synthesis kit (Vazyme, China). Real-time PCR was conducted using SYBR-Green fluorescence-based assays for signal detection (Vazyme) with complementary DNA. The housekeeping gene GAPDH was used as a reference for data normalization. The primer sequences used in the assay were as follows: For GAPDH, F: TGCACCACAACTGCTTAGC, R: GGCATGGACTGTGGTCATGAG. For CHEK2, F: TTGTCAAGAAGTTGTTGGTAGTGG, R: GTAGAGCTGTGGATTCATTTTCCT.

### Single-gene for gene-set enrichment analysis (GSEA)

To analyze potential pathways related to CHEK2 expression, we utilized the TCGA, ICGC, and GAO et al. datasets. GSEA was employed to generate an ordered list of all genes based on their correlation with CHEK2 expression in each dataset [44]. The reference gene sets used for analysis included KEGG, GO, Hallmarks, and Reactome [45–47]. We set the default threshold as |normalized enrichment score (NES)| ≥1, taking into account both nominal *P*-value and FDR q-value. The filter criteria for enrichment were set as nominal *P*-value < 5% and FDR *q*-value < 25%. Subsequently, we compared gene enrichment differences between the high-level CHEK2 group and the low-level CHEK2 group using GSEA Version 4.0.3 software.

### Constructed sgRNAs and established stable cell lines

We obtained and constructed sgRNAs targeting CHEK2 from Transheep Bio (Shanghai, China). To establish stable cell lines, HCCLM3(LM3), Huh7, and hepG2 cells were transfected with external 1 µg/mL of puromycin (ApexBio). Stable expression cell lines were successfully generated. Cloned sgRNA sequences used are as follows: sgCHEK2^1#^: AAGGGCCCATAATCGAGCCC; sgCHEK2^2#^: CTGCCCCCTGGGCTCGATTA.

### Western blot procedure

The transfected cells were treated by the previously described method [48]. Several primary antibodies were listed: CHEK2 (#3440 S, CST, USA), β-actin (#81115-1-RR, Proteintech, China), H2A.X (#10856-1-AP, Proteintech), BAX (#41162, CST), cytC (#12245-1-AP, Proteintech), MDM2 (#66511-1-Ig, Proteintech), CyclinD1 (#60186-1-Ig, Proteintech), P53 (#60283-2-Ig), Cleaved PARP (#5625 S, CST), BCL2 (#15071 S, CST), AMPK (#5832, CST) and Phospho-AMPKα (Thr172, #50081, CST).

### Immunohistochemistry (IHC)

IHC was performed according to the previous protocol [49]. Paraffin-embedded slides were incubated with the antibodies mentioned above. For the detection of the primary antibody, an avidin-biotin-peroxidase complex was employed. Anti-digoxigenin-HRP (anti-DIG HRP) antibodies and DAB were employed for the detection process. The staining intensity was assessed manually by two experienced pathologists, who scored the presence of brown-stained lymphocytes as positively stained cells. The IHC scores were calculated based on the average proportion of positively stained cells observed in five random fields.

### Colony formation assay

Transfected cells were seeded in culture dishes and incubated for 24 h. Subsequently, after removing the culture media, cells were grown in 4 ml of DMEM supplemented with 5% FBS until visible cell colonies formed. Colony counting was performed after staining with crystal violet using a light microscope.

### Edu assay

An Edu labeling assay was conducted using the Edu Kit (#C0075S, Beyotime, China) following the manufacturer’s protocol. After incubation, the cells were stained with DAPI for 10 min. Edu-positive cells were then captured using a suitable imaging technique.

### Cell cycle analysis

After being fixed by spending the night at –20 °C in 70% ethanol, the cells underwent three cold PBS washes. Following the manufacturer’s recommendations, the samples were then stained with PI/RNase Staining Buffer (BD Pharmingen, Franklin Lakes, NJ, USA) for 15 min at room temperature. A BD FACSCanto II Flow Cytometer was used to evaluate the labeled cells. Software called ModFit LT 3.1 was used for data analysis.

### SA-β-gal staining

Following the manufacturer’s instructions, cells were stained for the presence of SA-galactosidase using the Senescen-galactosidase Staining Kit (#C0602, Beyotime). Cells were briefly rinsed in PBS and then fixed for 15 min in the fixative solution. They were then treated with the staining solution for an overnight period at 37 °C without CO_2_. Finally, using an inverted microscope, green-stained positive cells were viewed and counted from three distinct positions within each well.

### Apoptosis assessment

The collected and washed transfected cells were incubated sequentially with Annexin-V-FITC (BD, USA; 10 μL, 15 min) and propidium iodide (PI; 5 μL, 5 min protected from light). Subsequently, flow cytometry was used for analysis.

### The mutational landscape in the low and high CHEK2 groups

The GISTIC2 module on the website (https://clue.io/) was utilized to obtain copy number variation data from the TCGA-LIHC database. The mutational landscape was then analyzed using R packages “MOVICS” and “maftools“ [50, 51].

### MitoSOX detection

HCC cells were labeled with MitoSOX (M36005, Invitrogen, USA) to examine mitochondrial production of reactive oxygen species (ROS). Briefly, 1 × 10^5^ cells were suspended in 100 μL of pre-warmed HBSS. Then, 100 μL of MitoSOX (20 μM) diluted in pre-warmed HBSS was added to each well. At 37 °C, the cells were incubated for 20 min. The stained cells were examined using a BD FACSCanto II Flow Cytometer following incubation.

### Detection of ATP and NAD/NAD^+^ ratio

For analysis of mitochondrial function, HCC cells were seeded in a 24-well plate and treated with an ATP assay kit (#S0026, Beyotime) and NADH^+^/NADH ratio assay kit (#S0175, Beyotime). Following the instructions provided, the samples were subsequently analyzed using a microplate reader.

### Electron microscopy

HCC cells that had been transfected were fixed for 2 h in a post-fixative solution made of 0.1 M sodium cacodylate buffer, 0.8% potassium ferrocyanide, and 2% osmium tetroxide. The samples were dehydrated in several acetone baths before being implanted in Embed 812 resin. After that, uranyl acetate and lead citrate were used to cut and post-stain ultrathin slices. Using a JEOL 1200EX transmission electron microscope, images and observations of the samples were taken at random.

### Cell-derived tumor xenograft (CDTX) for HCC

We initially established a xenograft HCC model in male nude mice. Every 5 × 10^6^ LM3/control, LM3/shCHEK2^1#^, LM3/shCHEK2^1#^ + Nultin-3 cells were inoculated into the right axillary subcutaneous area. The nude mice were euthanized after 4 weeks, and the tumors from each group were taken out. Then, using a cannula needle, the tumors were implanted into the livers of 8-week-old mice, who were then uniformly put to death after 2 weeks. All mice were randomly assigned to the experiments.

### Statistical analysis

All the statistical data analyses were carried out and generated via R software 4.1.3, all the plots were produced from R package “ggplot2”. All experiment’s quantitative data were given as mean SD. Student’s *t*-test or Wilcoxon test was used for data evaluation. Statistical significance was exhibited as follows: ns, not significant; *p < 0.05; **p < 0.01; ***p < 0.001, ****p < 0.0001.

### Supplementary information

supplementary figure legends

Full and uncropped western blots

SupFig1: CHEK2 could be a potential drug target for HCC

SupFig2: Knockout of CHEK2 selectively induces proliferation arrest, cell cycle blockade, and senescence in HCC cells with TP53 mutation.

SupFig3: Combining Nultin-3 further induces cell cycle arrest and inhibits growth in CHEK2-inhibited HCC cells with TP53 mutation.

SupFig4: Knockout of CHEK2 triggers apoptosis in Nultin-3 treated HCC cells.

SupFig5: Combining Nultin-3 and knockout of CHEK2 exacerbates the loss of mitochondrial ATP in HCC.

## Data Availability

The results of this study are supported by the data available at the IEU database (https://gwas.mrcieu.ac.uk/datasets/ieu-b-4953/), eQTLgen database (https://eqtlgen.org/cis-eqtls.html), GWAS catalog (https://www.ebi.ac.uk/gwas/efotraits/EFO_0000182), TCGA (https://portal.gdc.cancer.gov), ICGC (https://dcc.icgc.org/), and GEO (https://www.ncbi.nlm.nih.gov/geo/).
