# Supplementary material for: CHEK2 knockout is a therapeutic target for TP53-mutated hepatocellular carcinoma
Source: Cell Death Discov. 2024 Jan 19;10:37. doi: 10.1038/s41420-023-01777-4 (PMC10799024; doi:10.1038/s41420-023-01777-4)
Supplement: Supplementary file 1 — supplementary figure legends [file 41420_2023_1777_MOESM1_ESM.docx]

**Supplementary Figure Legends**

SupFig1：CHEK2 could be a potential drug target for HCC. (A-C) The DFI, PFI and DSS of low and high CHEK2 level in the TCGA LIHC database.

SupFig2: Knockout of CHEK2 selectively induces proliferation arrest, cell cycle blockade, and senescence in HCC cells with TP53 mutation. (A) The protein level of CHEK2 in five HCC cell lines (97H, LM3, BEL-7404, Huh7 and hepG2). (B-D) The bar plot of Colony counts between the control, sgCHEK2^1#^ and sgCHEK2^2#^ groups in LM3, Huh7 and hepG2 cell lines. (E-G) The bar plot of cell cycle proportion between the control, sgCHEK2^1#^ and sgCHEK2^2#^ groups in LM3, Huh7 and hepG2 cell lines.

SupFig3: Combining Nultin-3 further induces cell cycle arrest and inhibits growth in CHEK2-inhibited HCC cells with TP53 mutation. (A, C) The bar plot of Colony counts between the control, Nultin-3 and sgCHEK2^1#^ + Nultin-3 groups in LM3, Huh7 cell lines. (E-G) The bar plot of cell cycle proportion between the control, sgCHEK2^1#^ and sgCHEK2^1#^ + Nultin-3 groups in LM3, Huh7 cell lines.

SupFig4: Knockout of CHEK2 triggers apoptosis in Nultin-3 treated HCC cells. (A, B) The bar plot of apoptosis ratios between the control, Nultin-3 and sgCHEK2^1#^ + Nultin-3 groups in LM3, Huh7 cell lines.

SupFig5: Combining Nultin-3 and knockout of CHEK2 exacerbates the loss of mitochondrial ATP in HCC. (A, B) The bar plot of cell cycle proportion between the control, sgCHEK2^1#^ + Nultin-3 and sgCHEK2^1#^ + Nultin-3 + Metformin groups in LM3, Huh7 cell lines. (C, D) The bar plot of Colony counts between the control, sgCHEK2^1#^ + Nultin-3 and sgCHEK2^1#^ + Nultin-3 + Metformin groups in LM3, Huh7 cell lines. (E, F) The bar plot of apoptosis ratios between the control, sgCHEK2^1#^ + Nultin-3 and sgCHEK2^1#^ + Nultin-3 + Metformin groups in LM3, Huh7 cell lines.
