# Supplementary material for: CHEK2 knockout is a therapeutic target for TP53-mutated hepatocellular carcinoma
Source: Cell Death Discov. 2024 Jan 19;10:37. doi: 10.1038/s41420-023-01777-4 (PMC10799024; doi:10.1038/s41420-023-01777-4)

Fig2

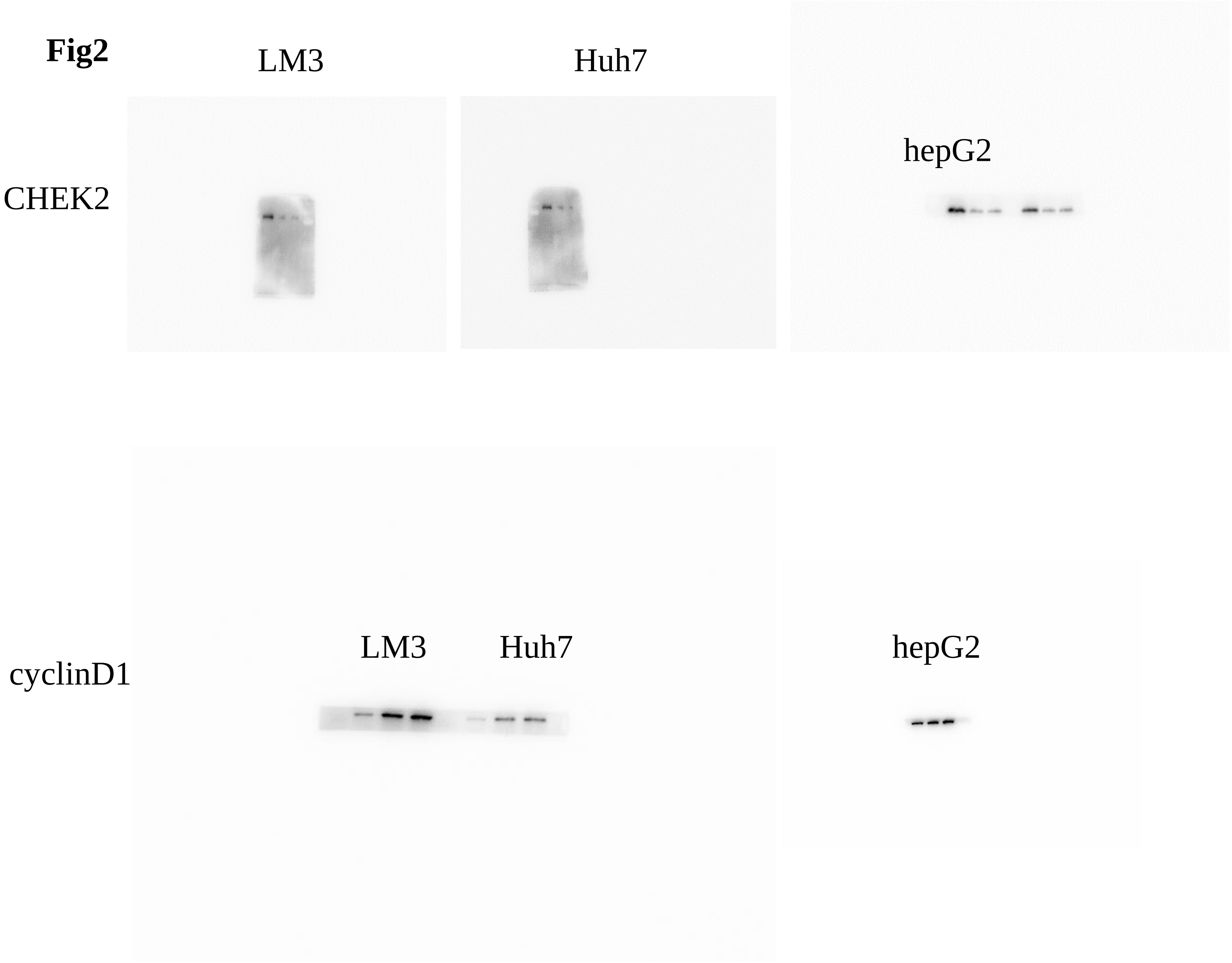

Fig2

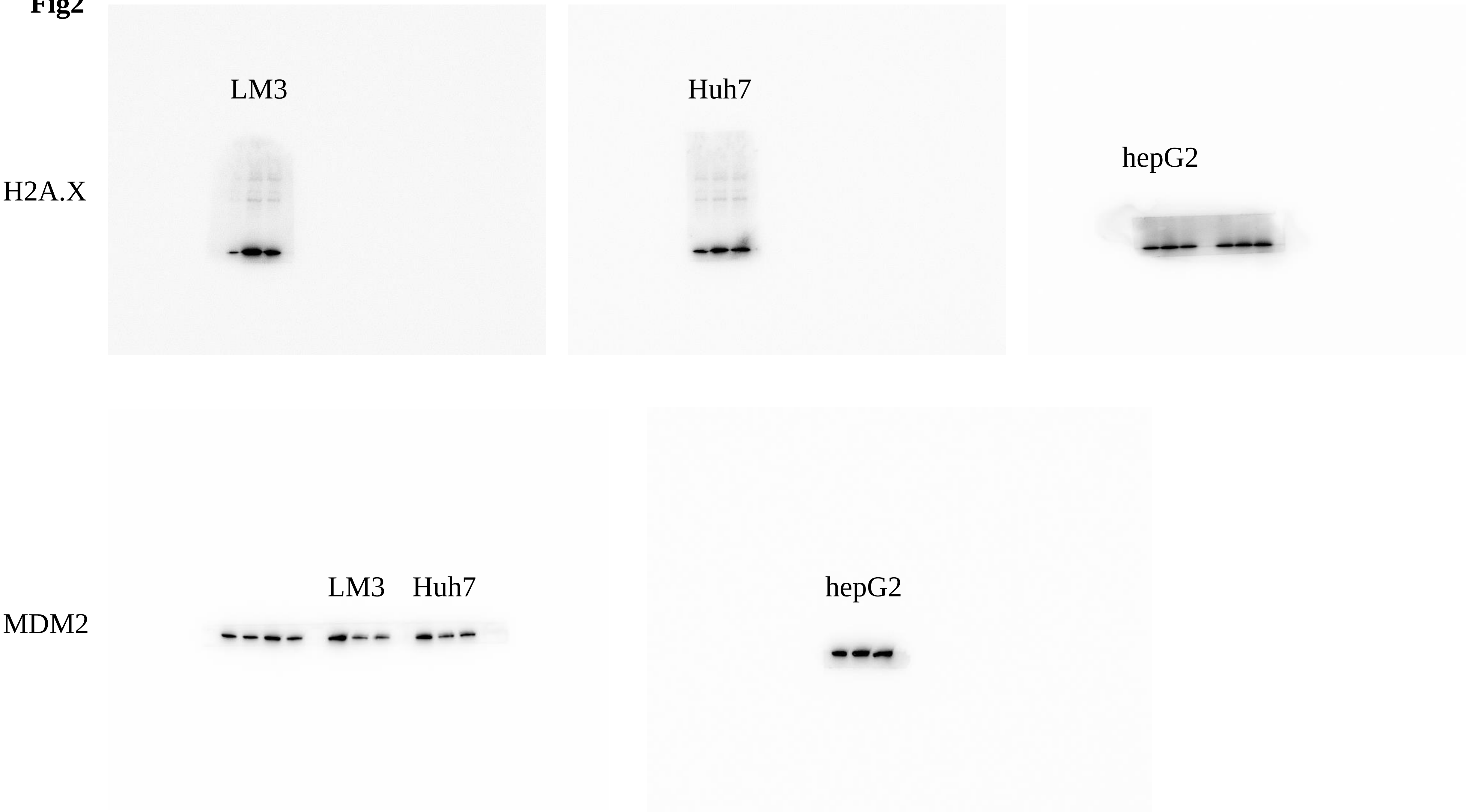

Fig2

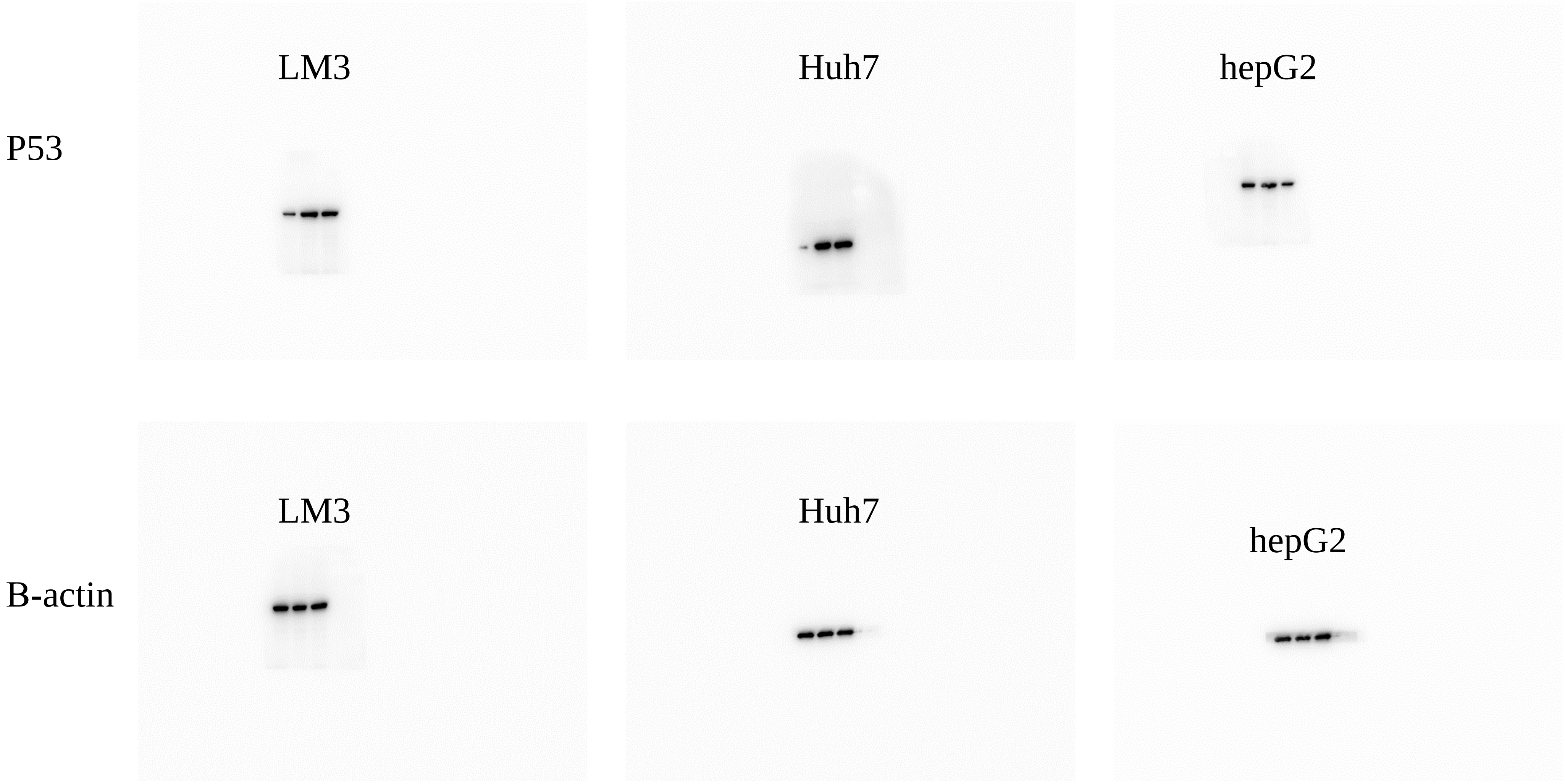

Fig3

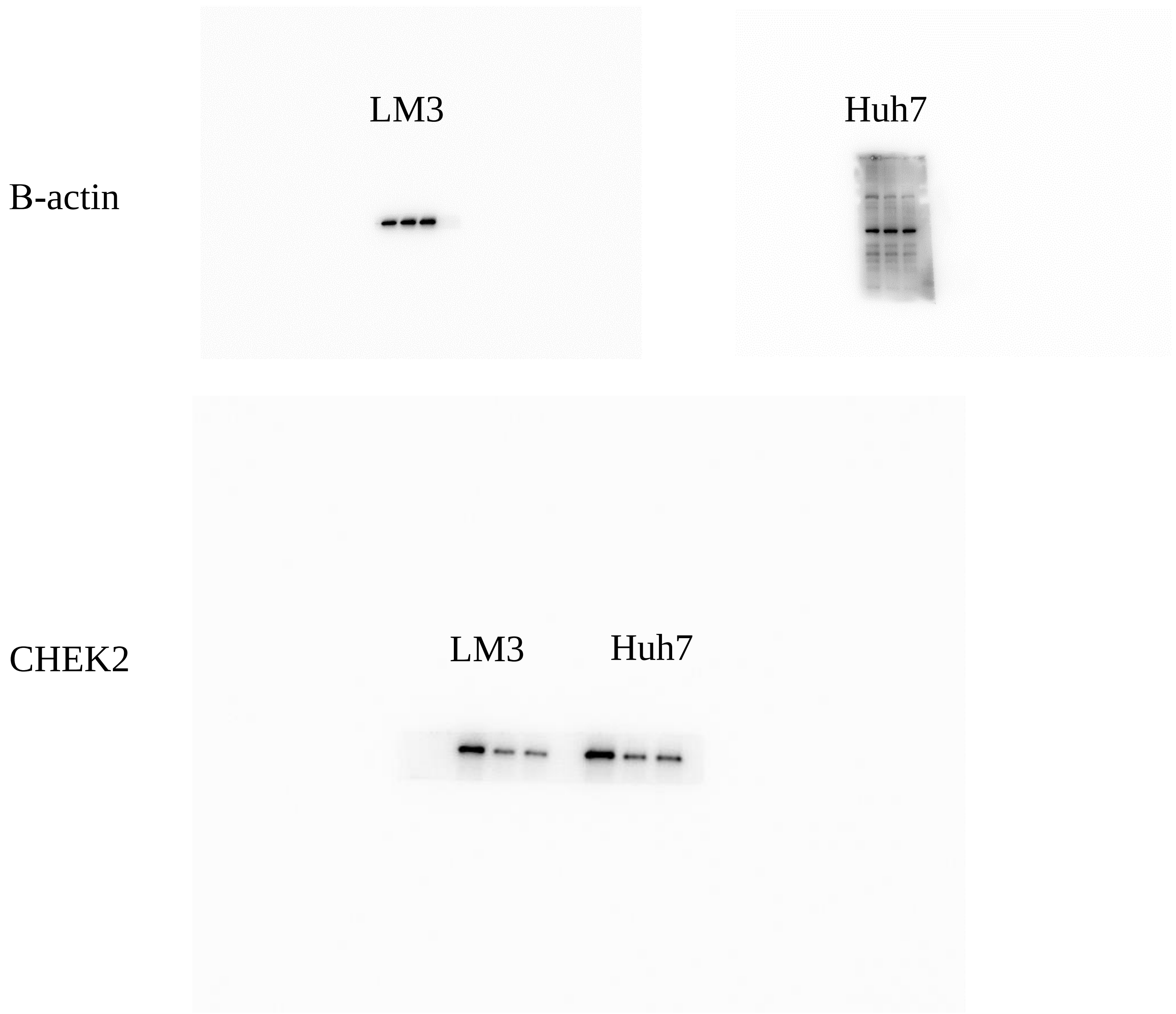

Fig3

cyclinD1

H2AX

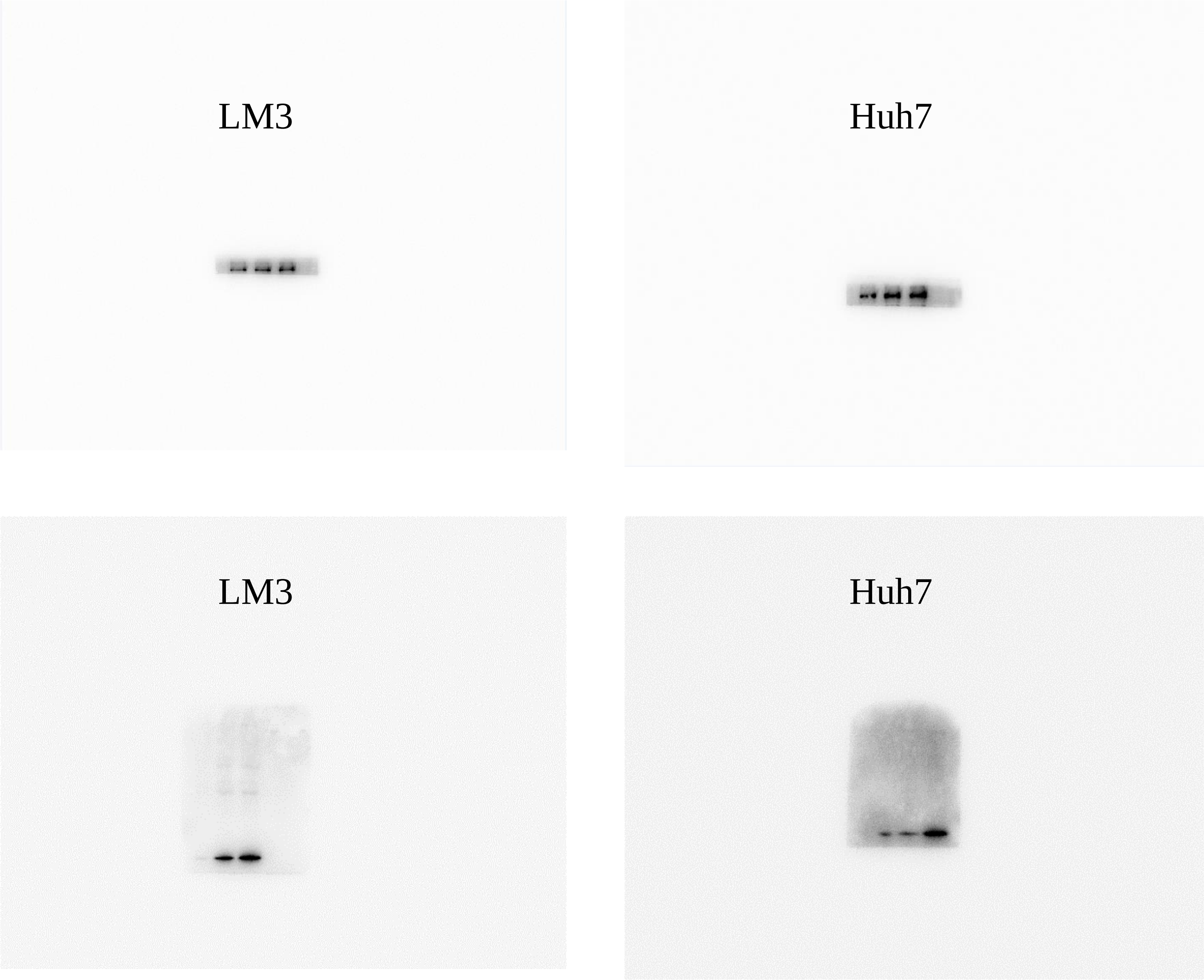

Fig3

MDM2

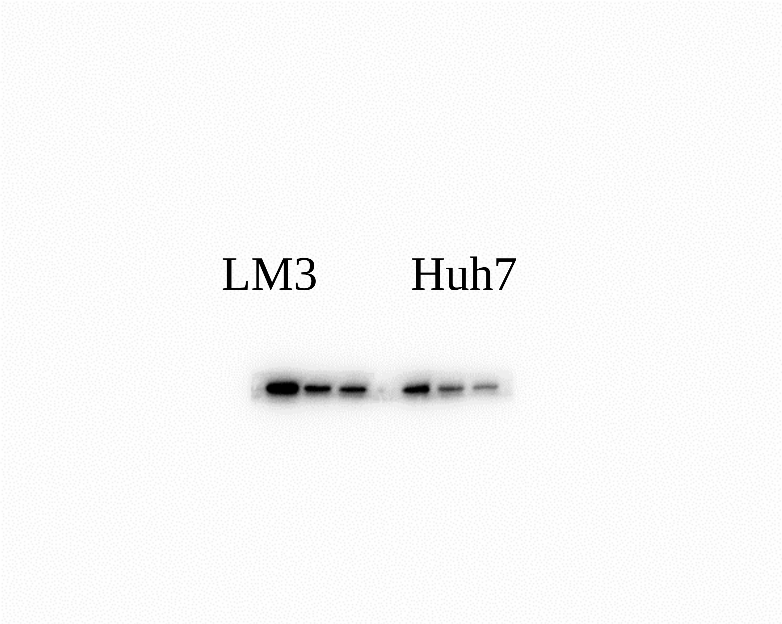

P53

LM3

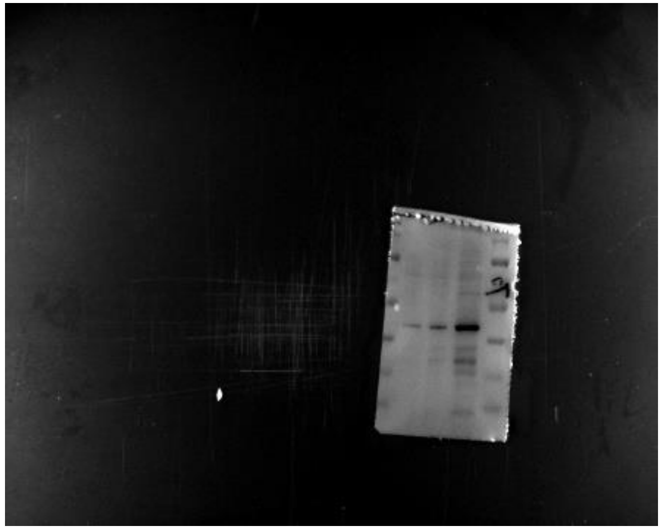

Huh7

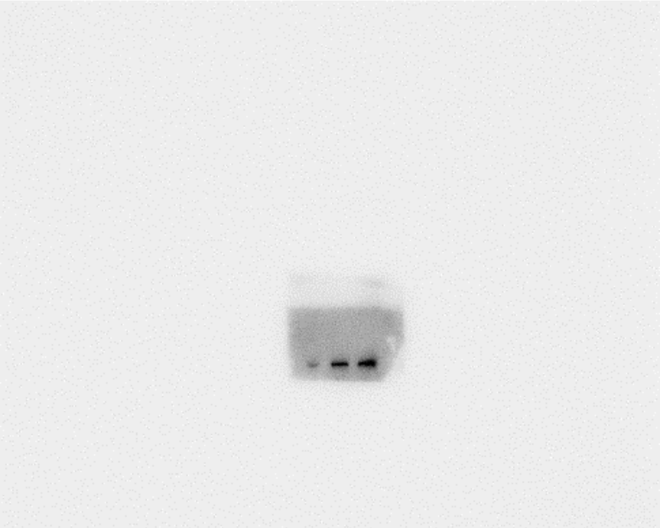

Fig4

LM3 Huh7

BAX

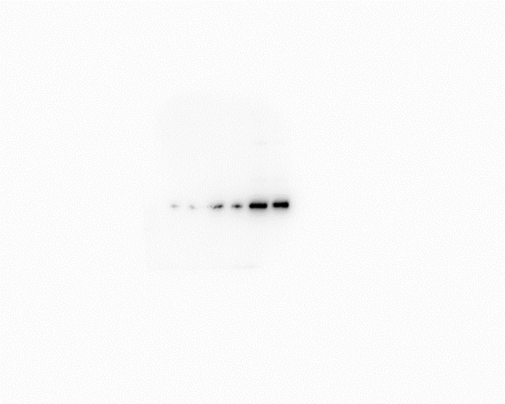

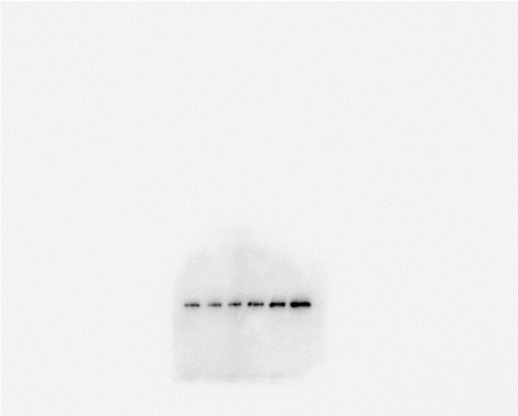

LM3 Huh7

BCL2

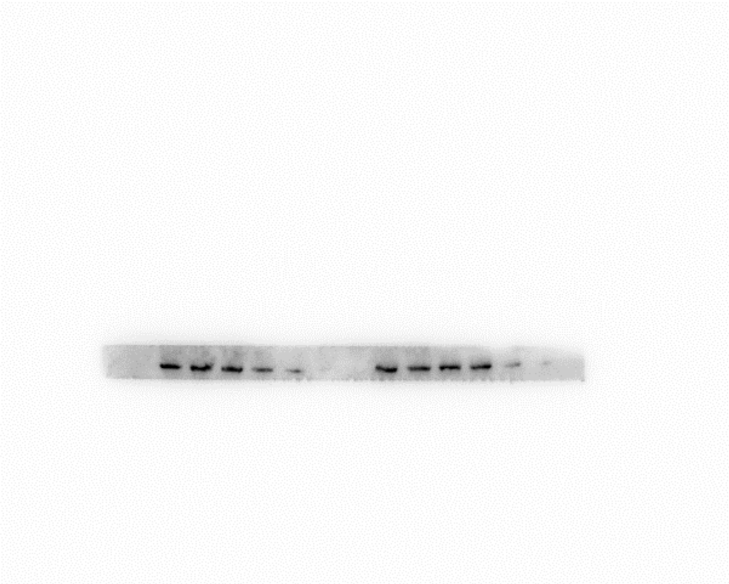

Fig4

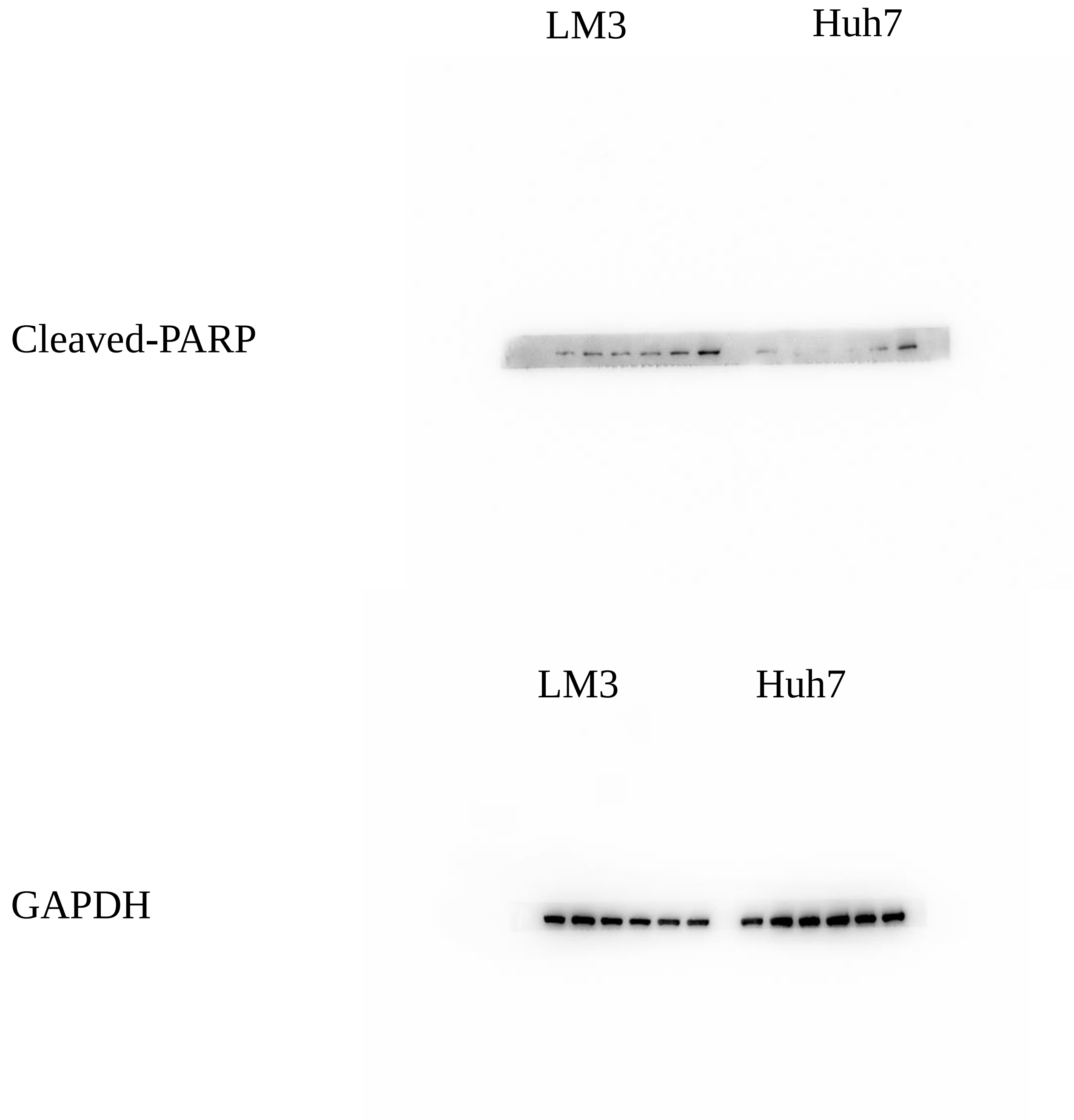

Fig5

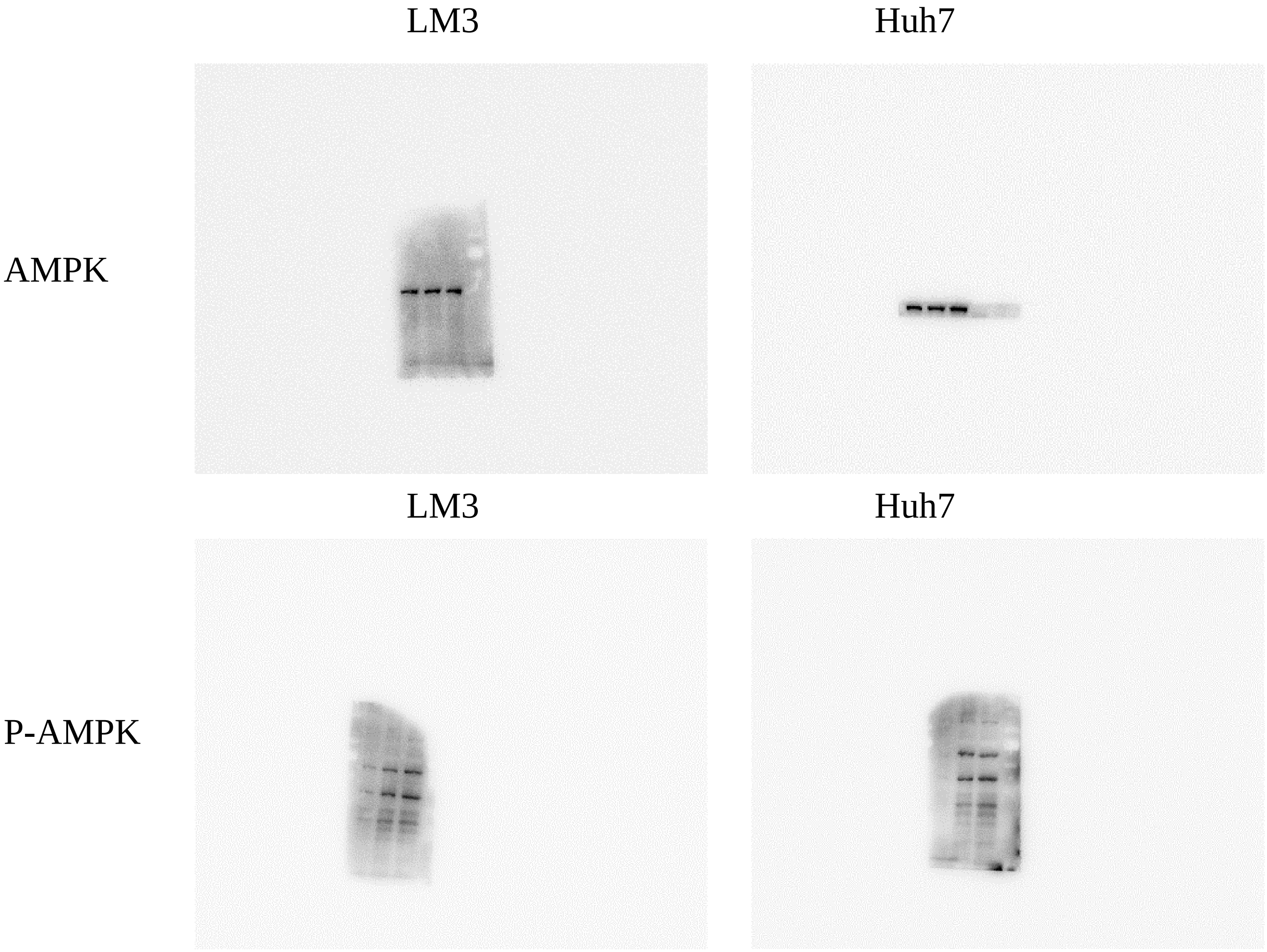

Fig5

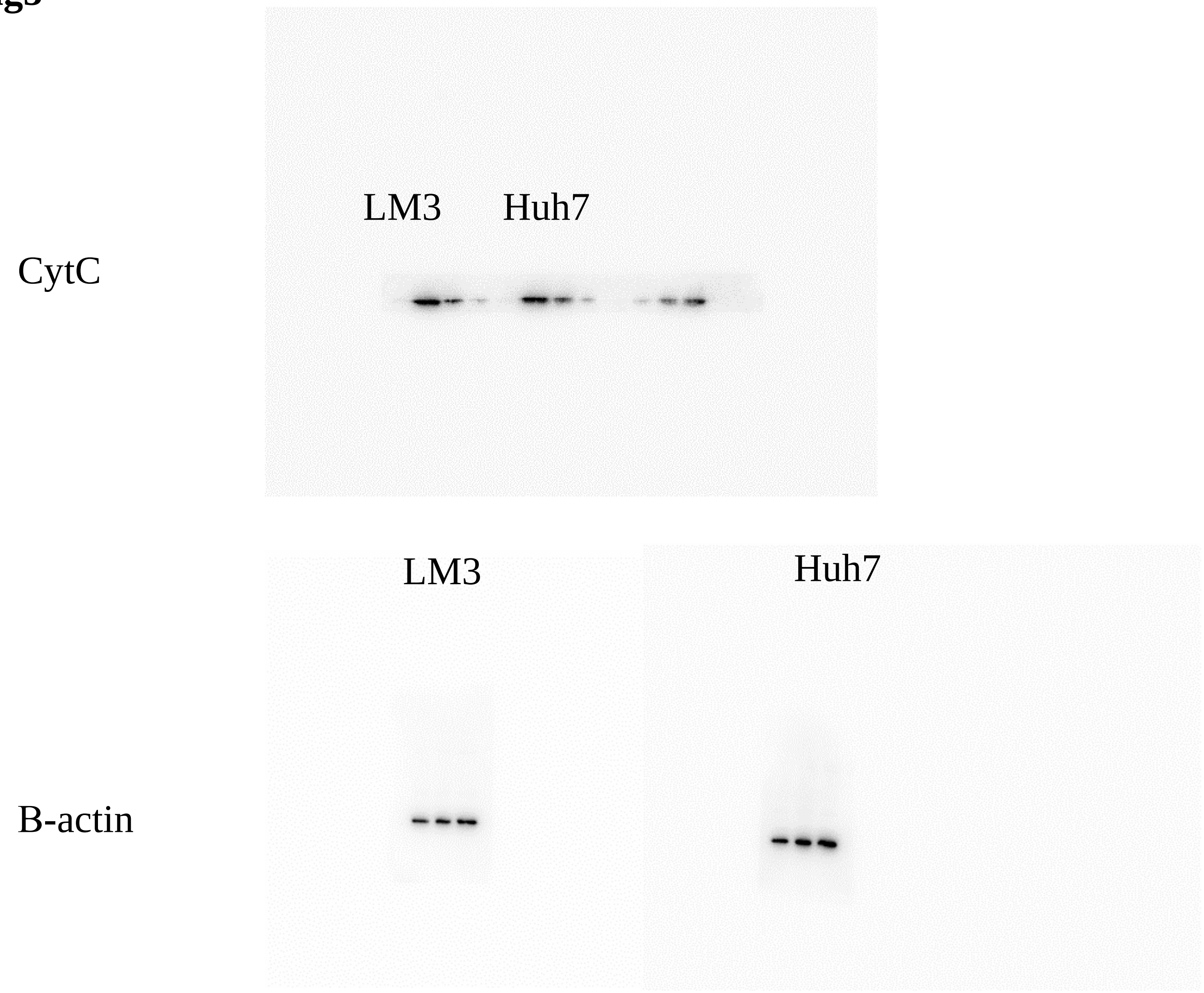

Fig6

LM3 Huh7

AMPK

LM3 Huh7

BAX

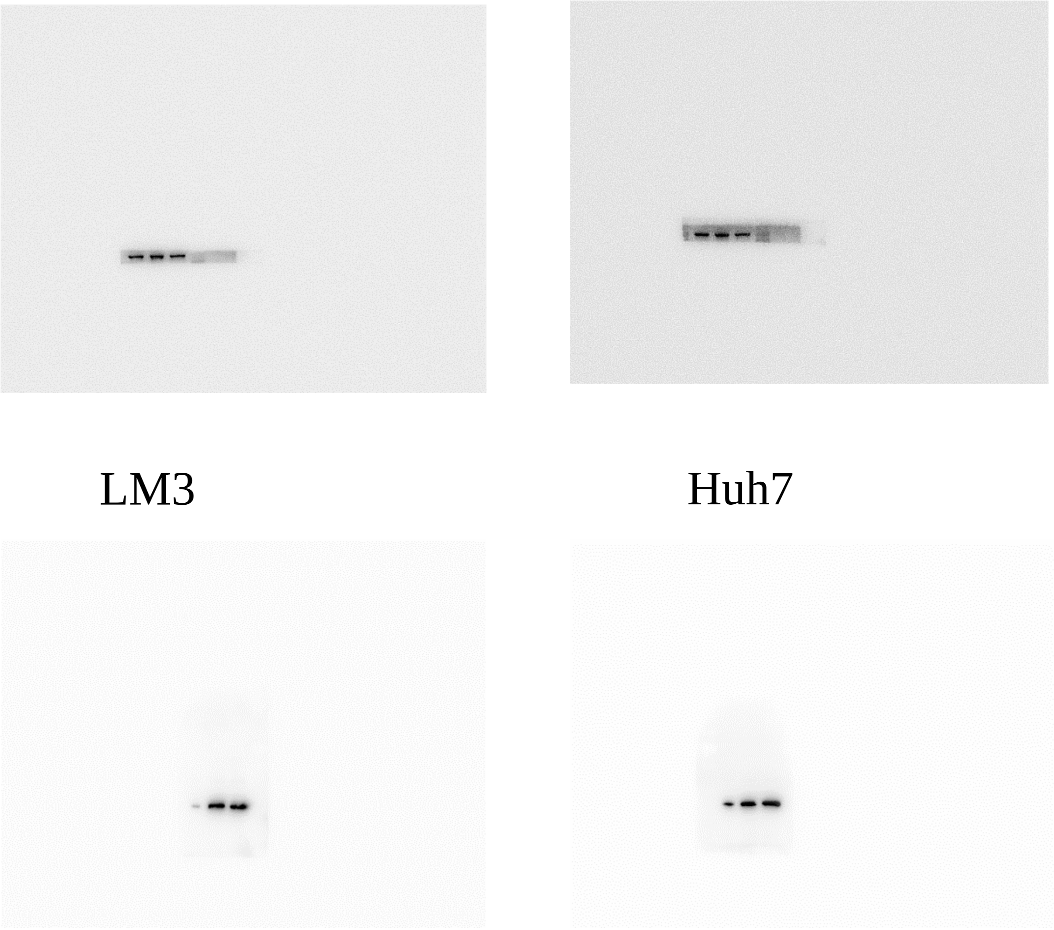

Fig6

LM3 Huh7

BCL2

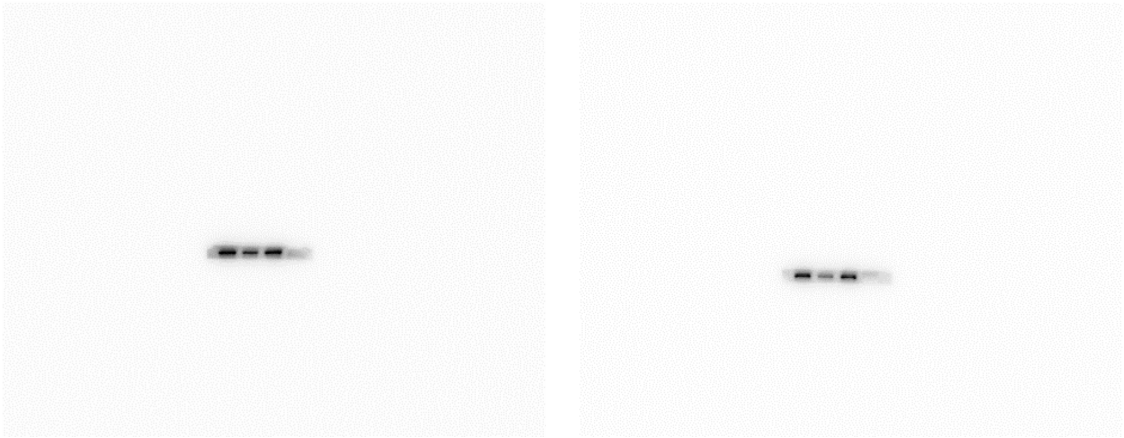

Cleaved-parp

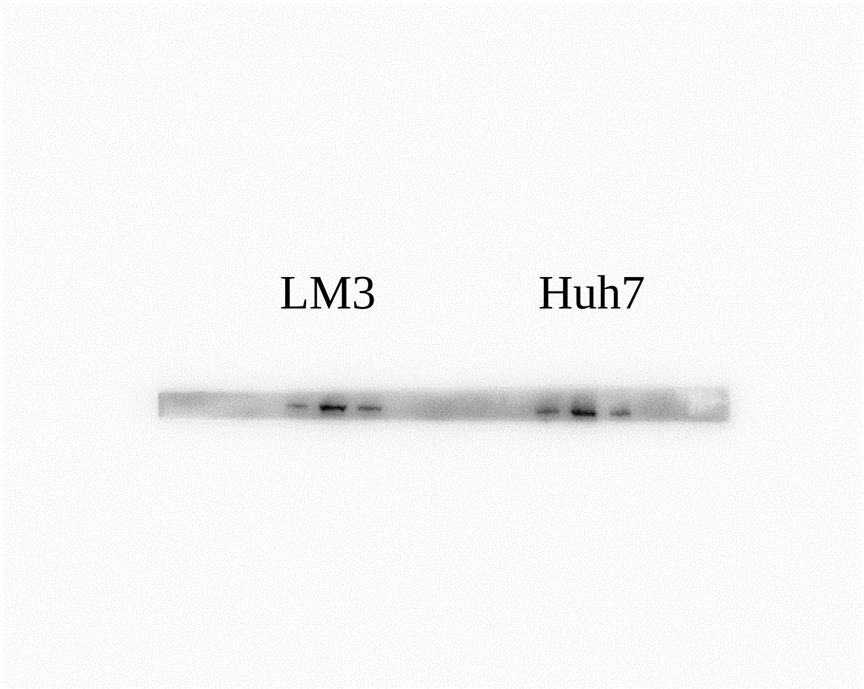

Fig6

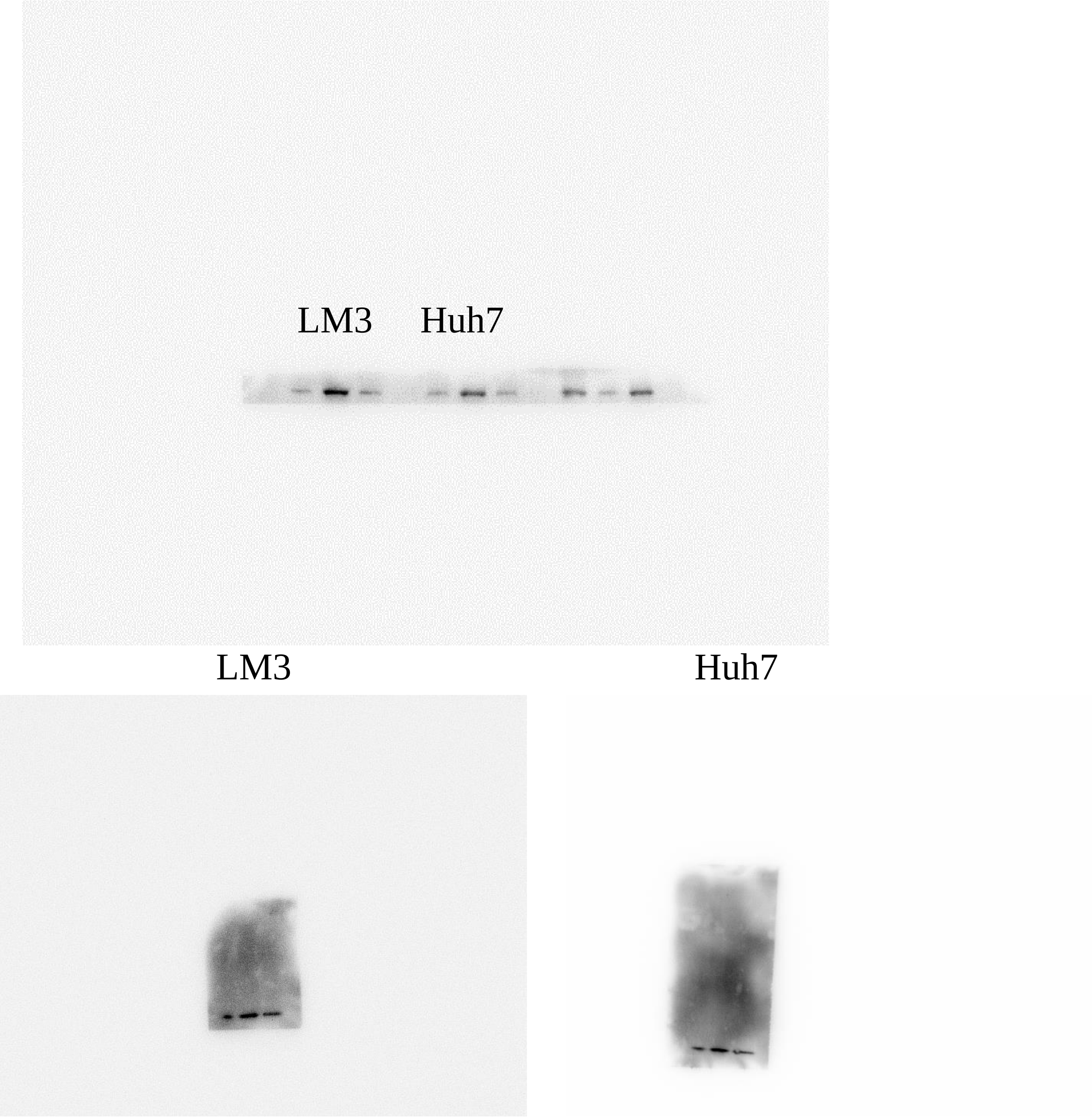

Fig6

LM3 Huh7

H2A.X

LM3 Huh7

MDM2

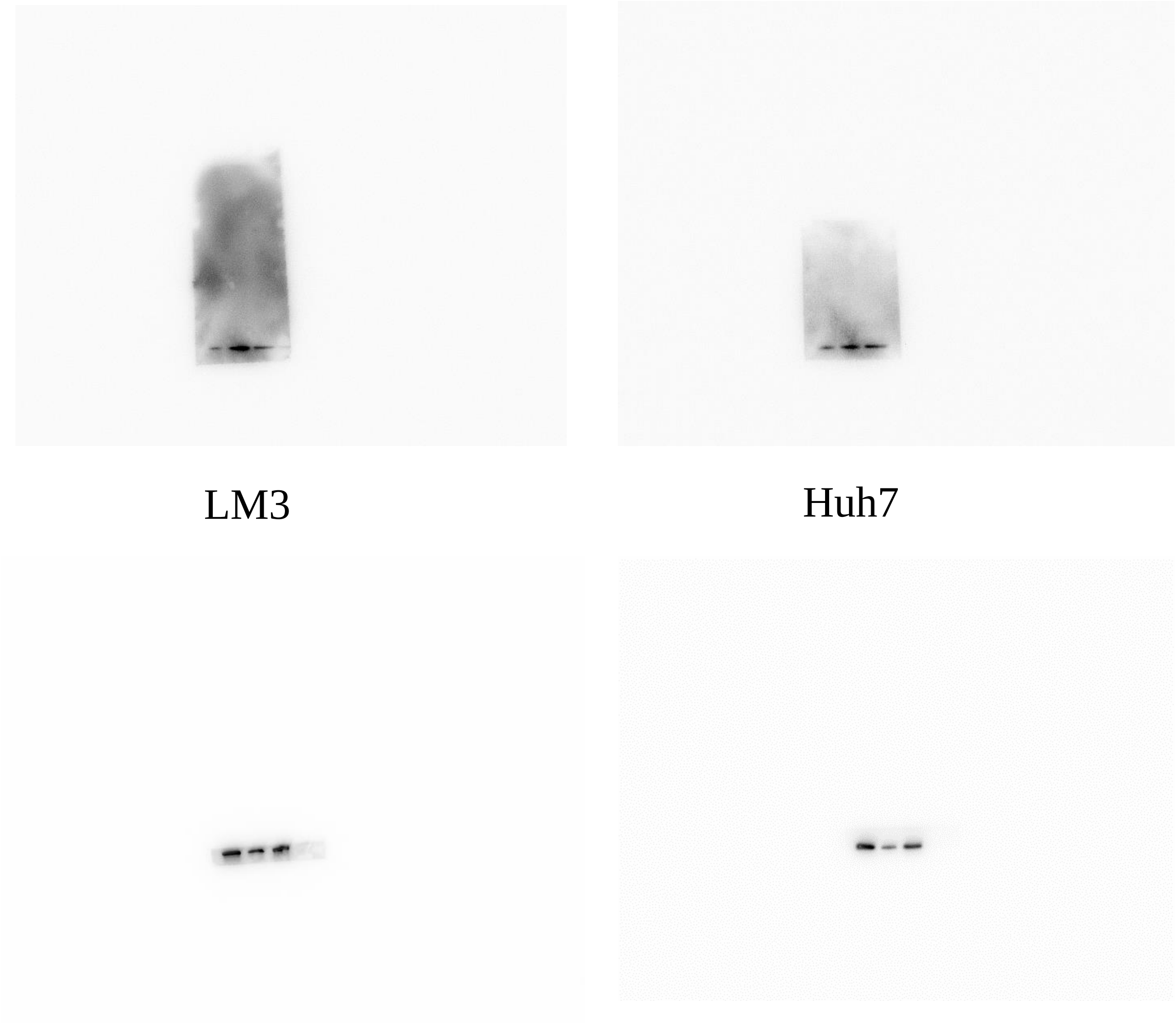

Fig6

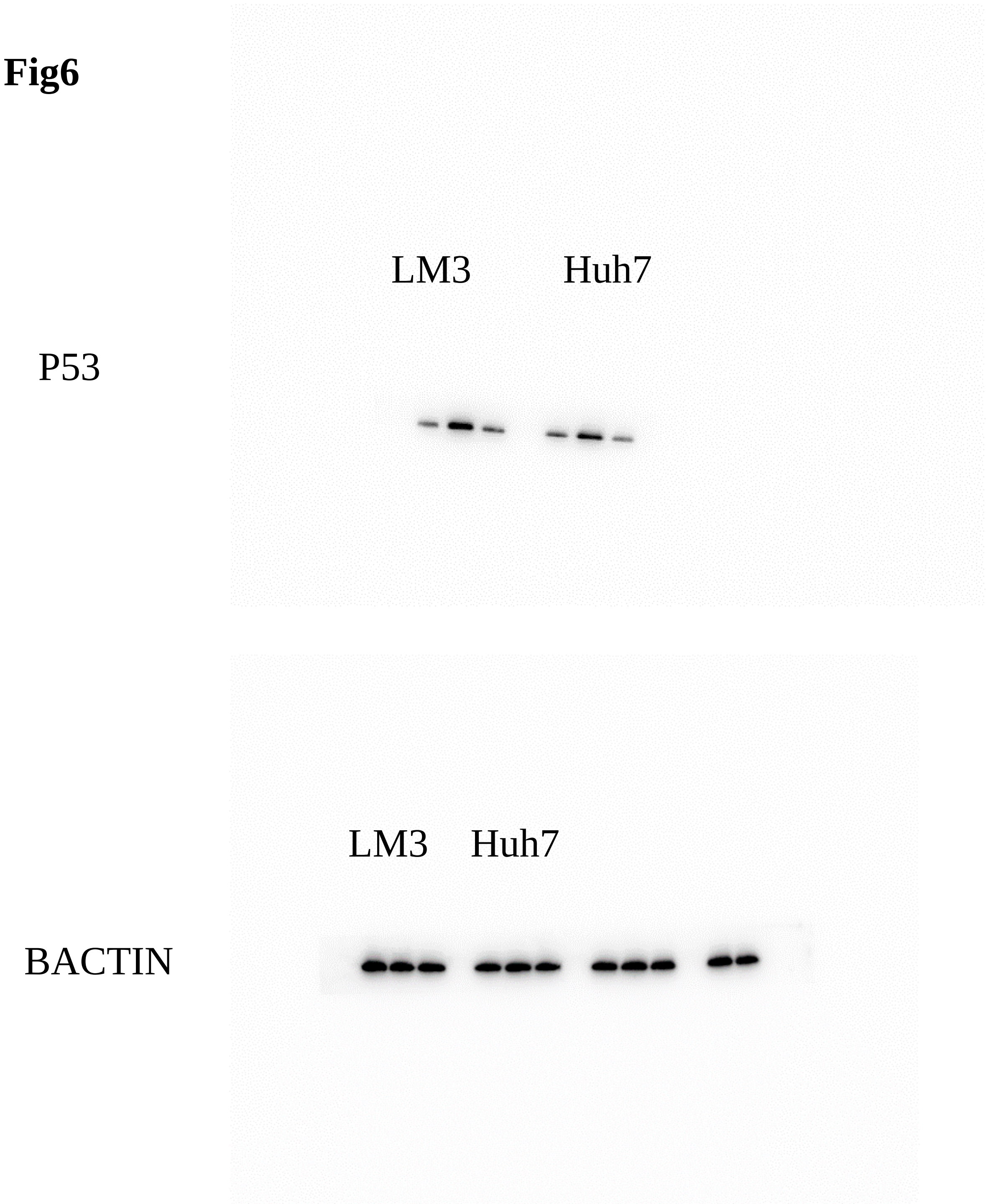

Fig6

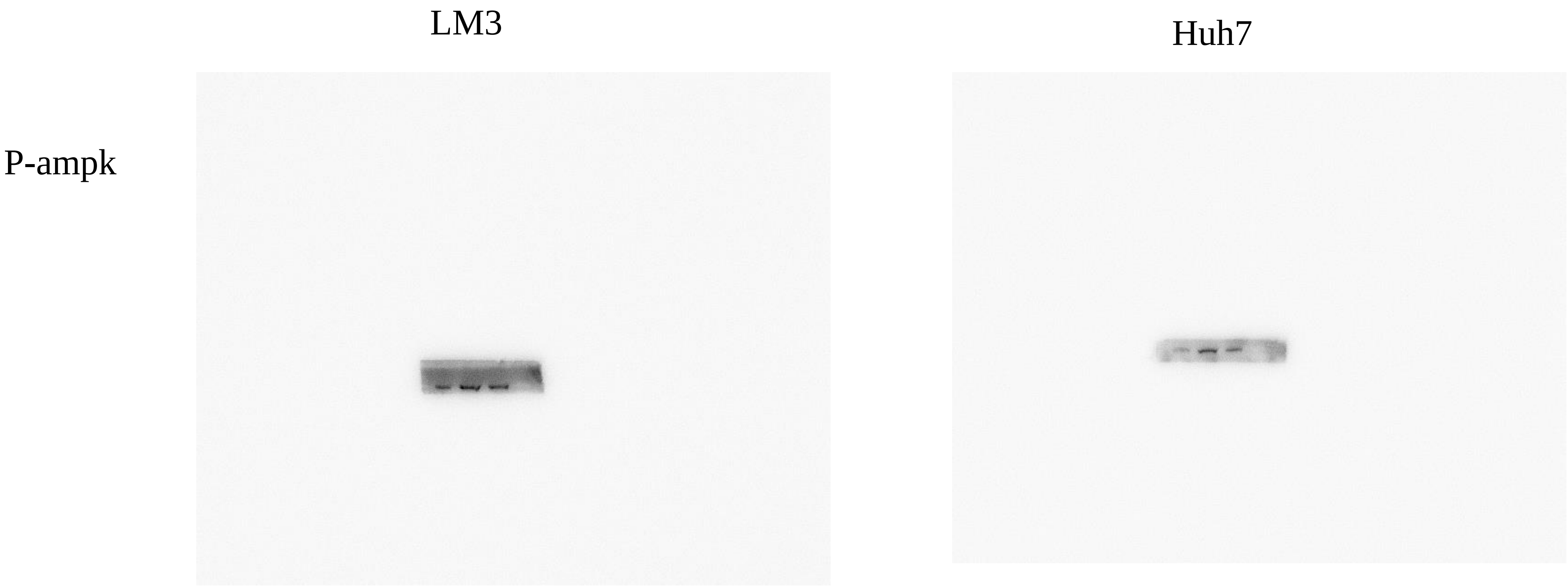

supFig2

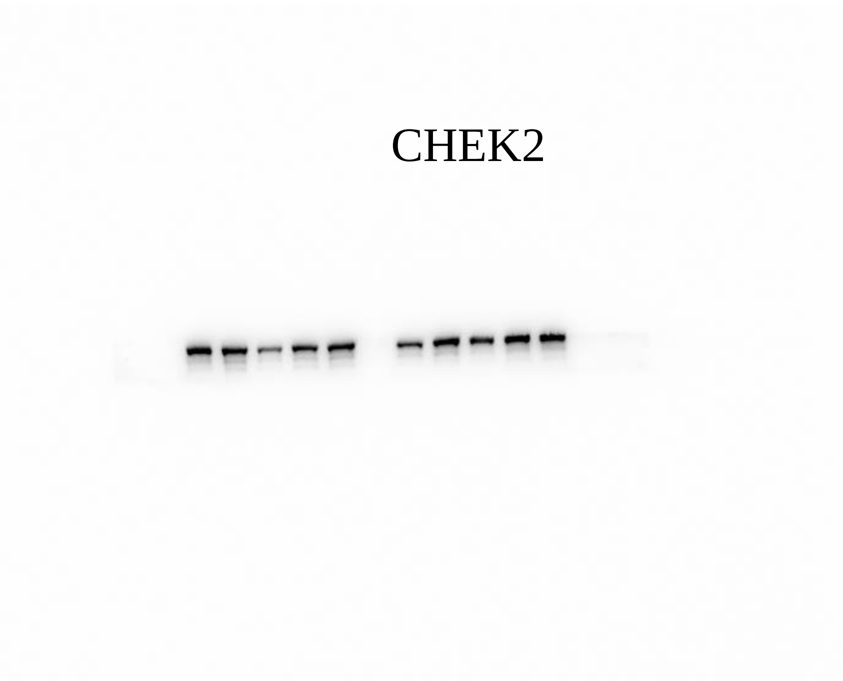

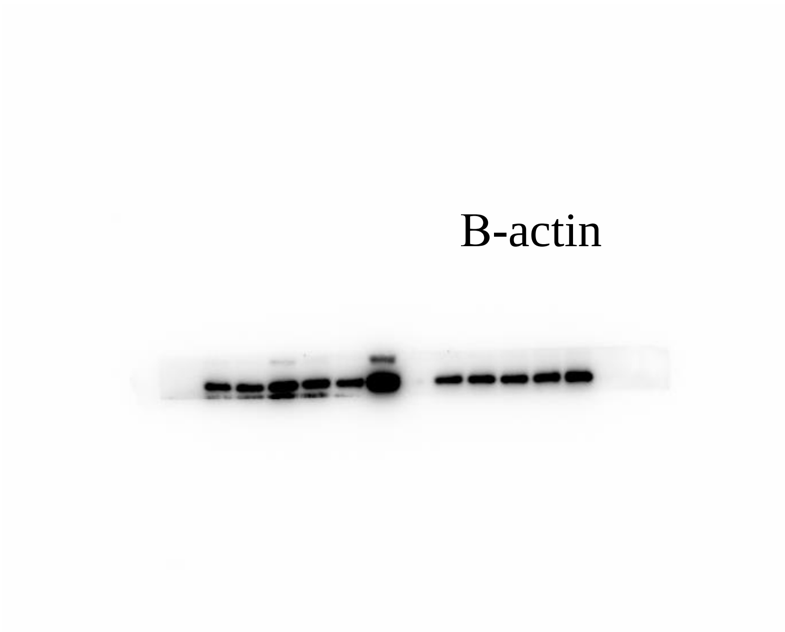

Supplement: Supplementary file 2 — Full and uncropped western blots [file 41420_2023_1777_MOESM2_ESM.pdf]
