# Supplementary figures and images for: CHEK2 knockout is a therapeutic target for TP53-mutated hepatocellular carcinoma
Source: Cell Death Discov. 2024 Jan 19;10:37. doi: 10.1038/s41420-023-01777-4 (PMC10799024; doi:10.1038/s41420-023-01777-4)

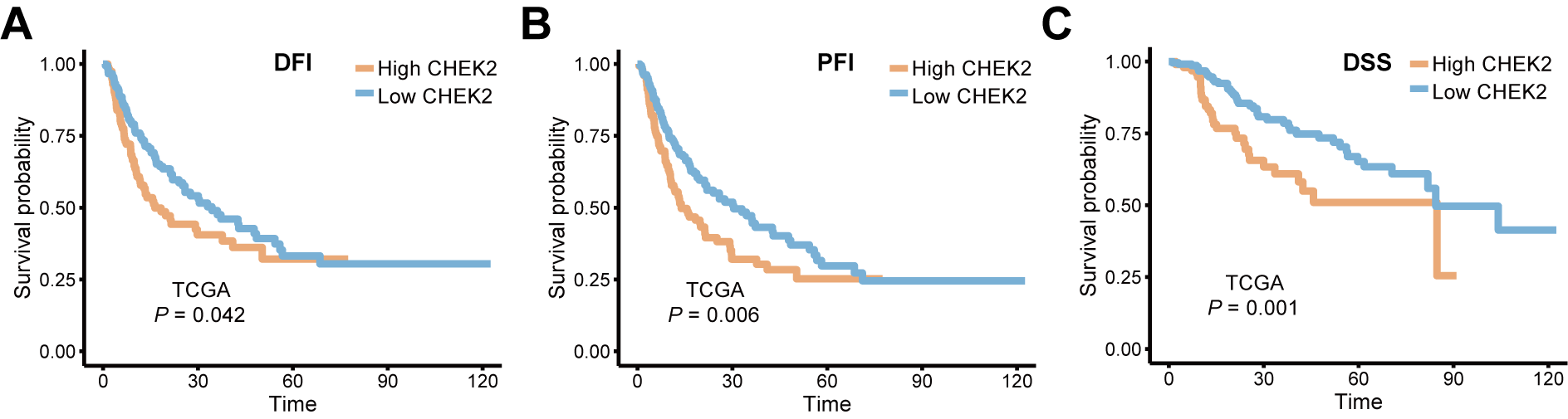

Supplement: Supplementary file 3 — SupFig1: CHEK2 could be a potential drug target for HCC [file 41420_2023_1777_MOESM3_ESM.tif]

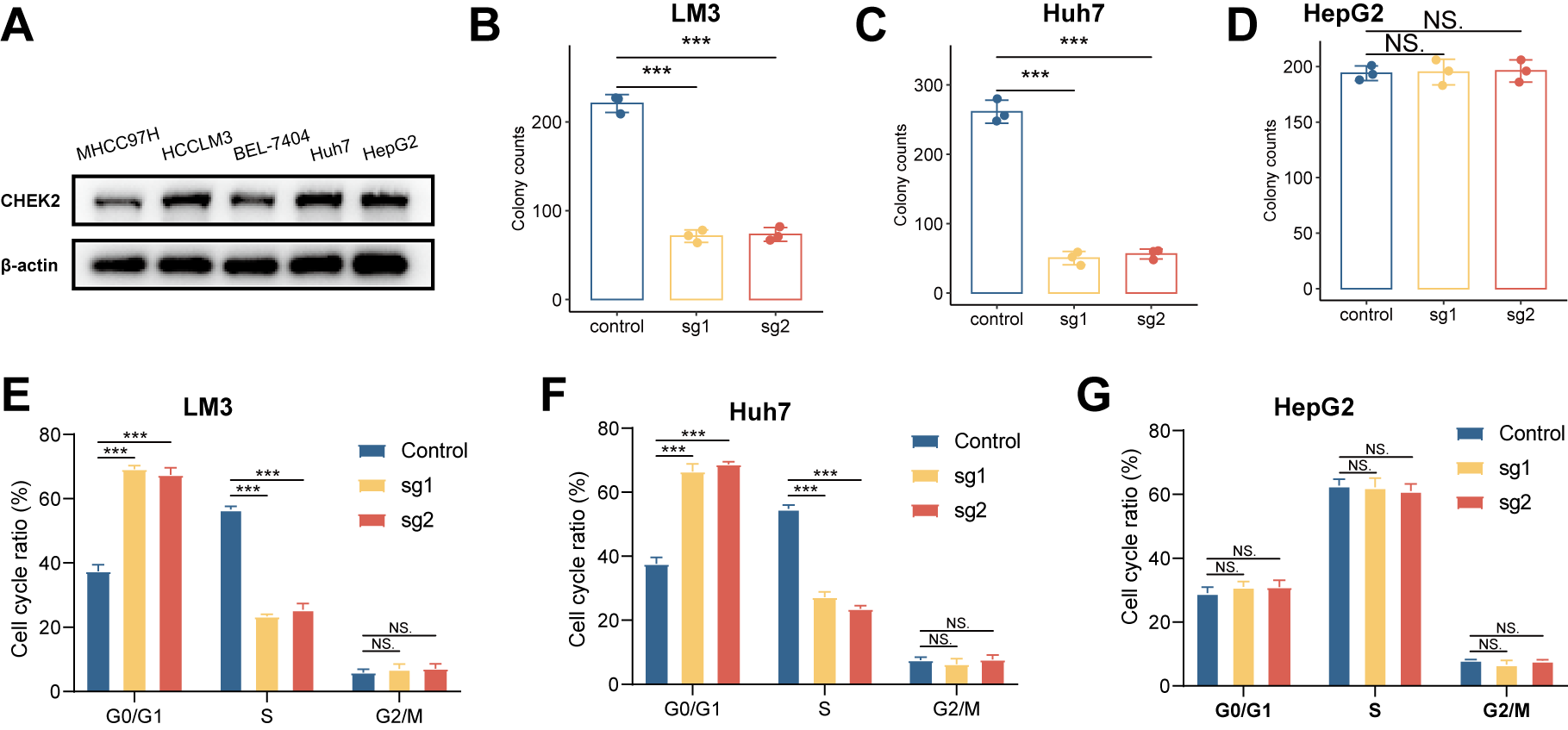

Supplement: Supplementary file 4 — SupFig2: Knockout of CHEK2 selectively induces proliferation arrest, cell cycle blockade, and senescence in HCC cells with TP53 mutation. [file 41420_2023_1777_MOESM4_ESM.tif]

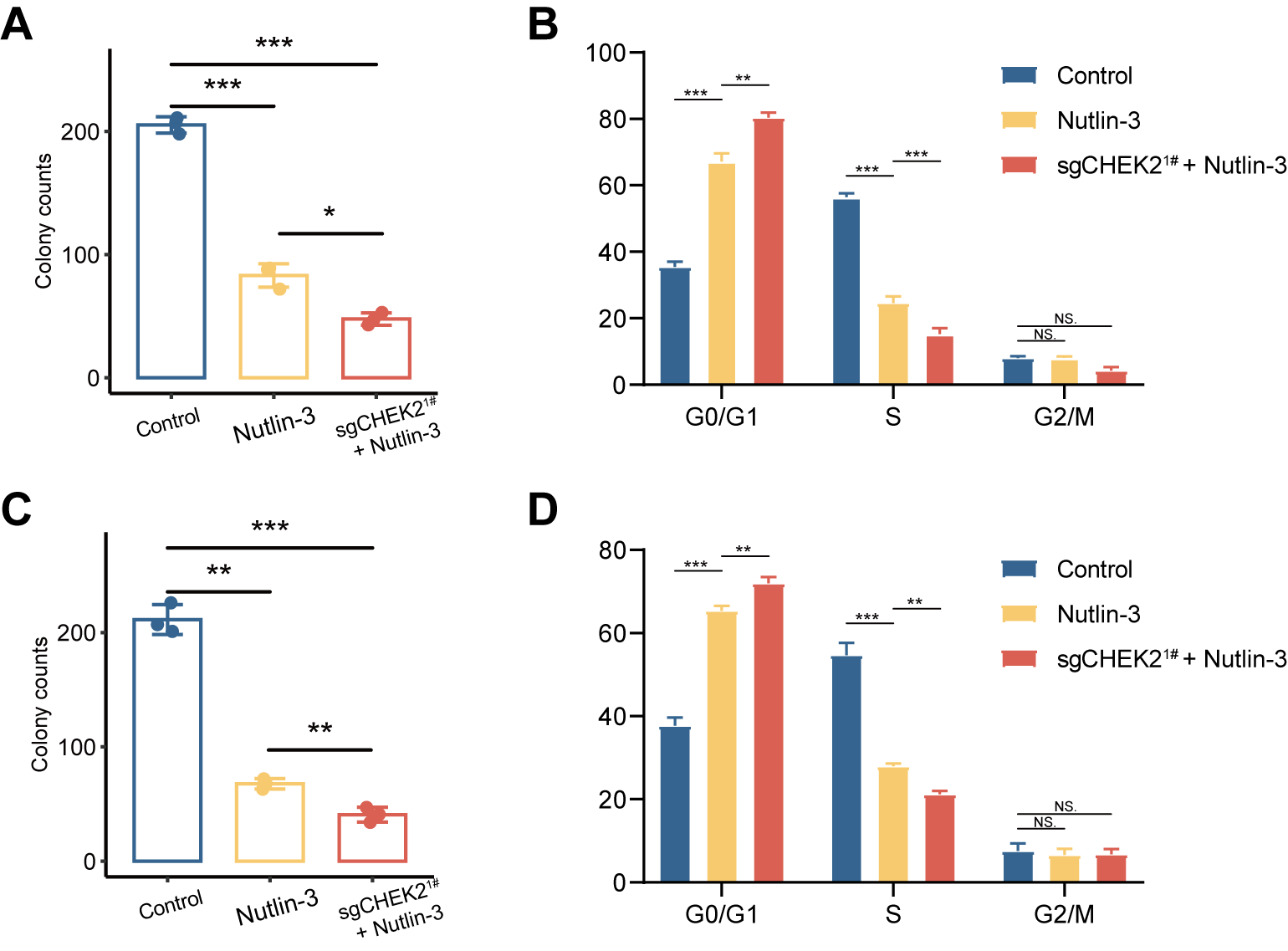

Supplement: Supplementary file 5 — SupFig3: Combining Nultin-3 further induces cell cycle arrest and inhibits growth in CHEK2-inhibited HCC cells with TP53 mutation. [file 41420_2023_1777_MOESM5_ESM.tif]

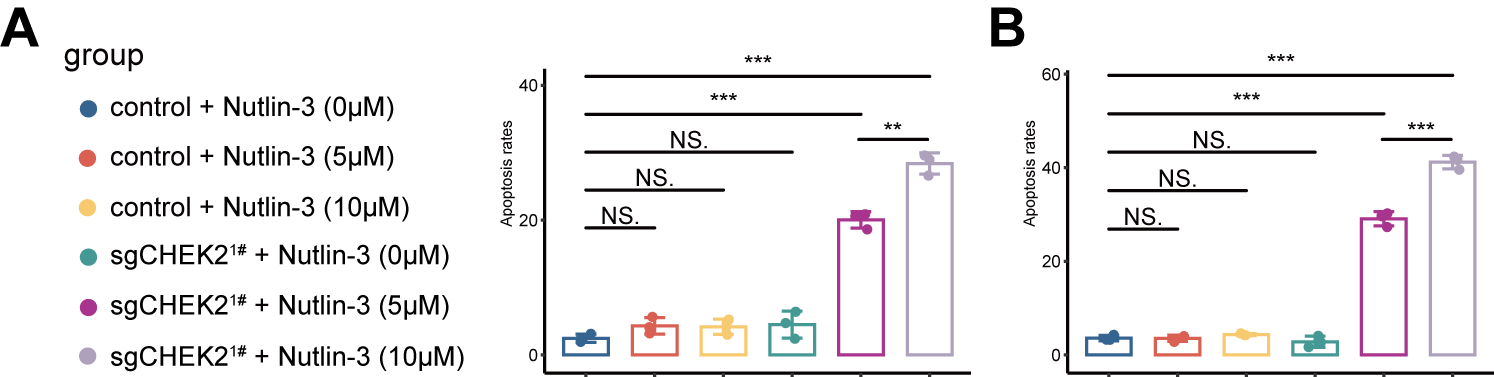

Supplement: Supplementary file 6 — SupFig4: Knockout of CHEK2 triggers apoptosis in Nultin-3 treated HCC cells. [file 41420_2023_1777_MOESM6_ESM.tif]

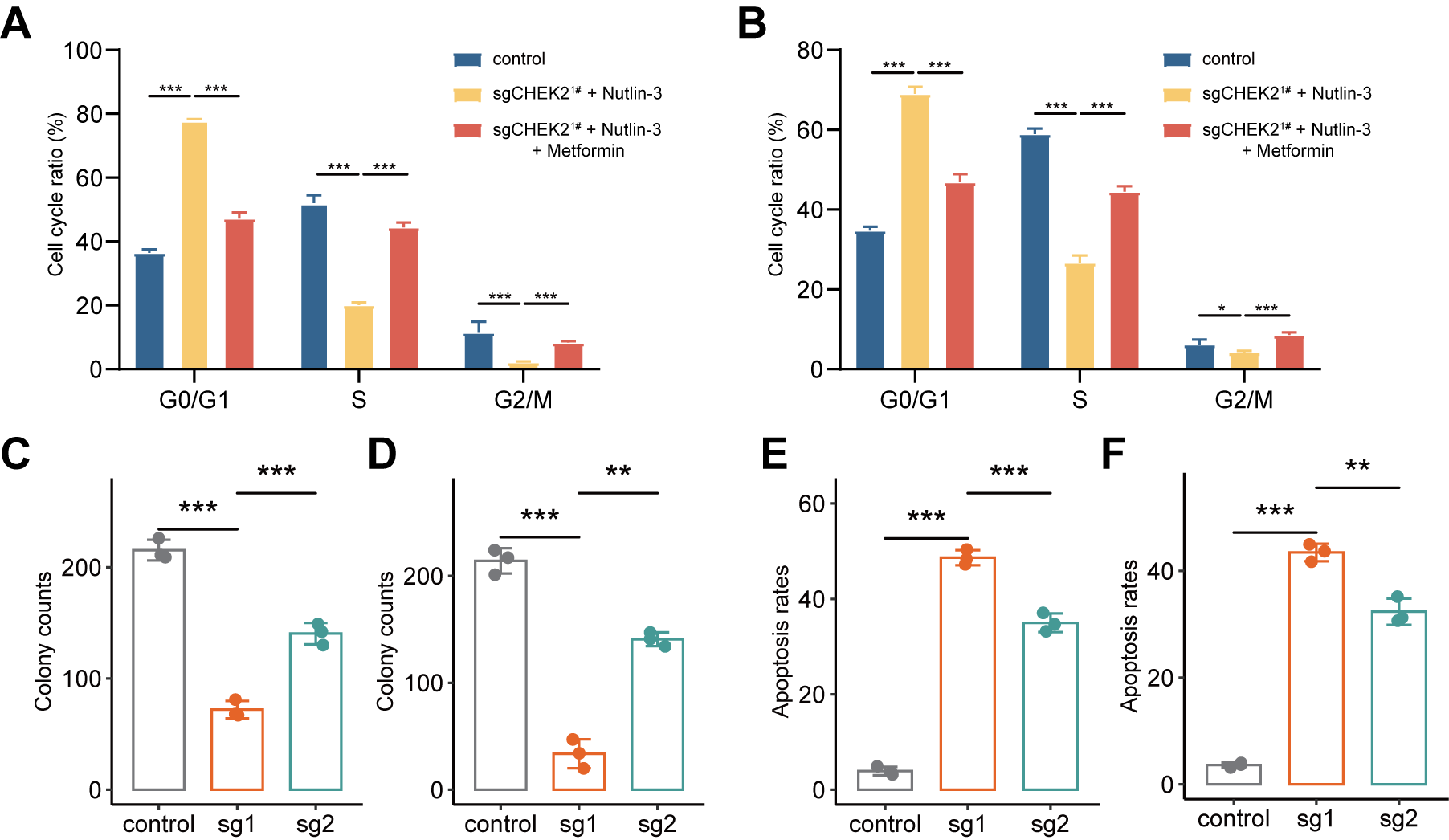

Supplement: Supplementary file 7 — SupFig5: Combining Nultin-3 and knockout of CHEK2 exacerbates the loss of mitochondrial ATP in HCC. [file 41420_2023_1777_MOESM7_ESM.tif]
